# Virtual Consultations for People With Intellectual Disabilities in General Practice and Community Care: Mixed Methods Qualitative Study

**DOI:** 10.2196/81173

**Published:** 2026-05-20

**Authors:** Freda Mold, Anna Cox, Vicki Tsianakas, Harm van Marwijk, Paul Shanahan, Treena Parsons, Jo Armes

**Affiliations:** 1 School of Health Sciences Faculty of Health & Medical Sciences University of Surrey Guildford United Kingdom; 2 Florence Nightingale Faculty of Nursing, Midwifery & Palliative Care King's College London London United Kingdom; 3 Department of Primary Care and Public Health Brighton and Sussex Medical School Brighton United Kingdom; 4 Your Healthcare CIC Surbiton United Kingdom

**Keywords:** access to care, patient safety, general practice, community care, digital inclusion, information and communication technologies, remote consultations, video conferencing, intellectual disabilities, virtual consultations

## Abstract

**Background:**

Virtual consultations (VCs) using video or telephone were embraced at speed in general practice (GP) and community care during the COVID-19 pandemic. People with intellectual disabilities, their families, and support workers (SWs), along with health care professionals (HCPs), had to adapt quickly to this change in provision, but little is known about how this new way of working was experienced.

**Objective:**

This study aims to explore the views and experiences of people with intellectual disabilities, their families, SWs, GP, and community care professionals on the quality and safety of VCs.

**Methods:**

This paper reports on users’ experiences of VCs, as part of a larger Experience-Based Co-design study. This paper relates to 2 stages of data collection. Observed video consultations in GP and community care (n=3), and semistructured interviews with people with intellectual disabilities, their family members or SWs, GP, and community care professionals (n=34). The 30-month study was conducted from November 2021 ending in April 2024. Data were analyzed using framework analysis.

**Results:**

Integrated results are presented through 5 themes, encompassed under an overarching theme of safety and quality. The five themes highlight critical factors in planning, delivery, and aftercare of VCs in GP and community services for people with intellectual disabilities in the United Kingdom: (1) context, space, and purpose—showing the importance of safe spaces to talk, and having clear consultation objectives and purpose; (2) choice—facilitating choice over time about modality of health care contact; (3) familiarity, online relationships, and trust—the building blocks for quality consultations; (4) prepare and personalize—to ensure that HCPs are aware of reasonable adjustments, and recognition of caregiver involvement; and (5) continued connection—where patients or families are offered continued contact with a named or same HCP enhancing access to regular or ongoing care. All participants were aware of the limitations of VC, which may impact safety, such as gaps in home monitoring due to the absence of appropriate equipment or recording, inability to identify vital risk indicators, and limited field of vision on screen. However, participants were also aware of the distinct benefits they offer in terms of quality provision, such as timeliness of care, building and sustaining comfortable relationships, support for more frequent attendance, and continuous connection to health teams.

**Conclusions:**

VCs offer an opportunity to improve digital inclusion in health care for people with intellectual disabilities. However, the quality and safety of VCs for this population are dependent on continuous review of patients’ needs over time and ensuring that their choices and preferences are considered when planning and providing ongoing care.

## Introduction

The post–COVID-19 general practice (GP) and community health care landscape has evolved significantly [1], with digital access now a key priority in health care systems, including the National Health Service (NHS) [2-4]. Recent guidance includes a range of online services, for example, online appointment booking, test results and record access, online prescription ordering, and telephone and video consultations [4].

Online service provision, such as virtual consultations (VCs) in GP and community care, has brought into sharp focus how health care provision might be adapted using new technologies to support widening access to diverse patient groups [5,6]. However, online services have potential limitations for both patients and service providers. Patients face challenges related to access, affordability, and the skills or support required, which may limit their ability to navigate and use these services effectively [7-9]. For health care professionals (HCPs), providing online services can impact their confidence and skills in using different modes of remote care delivery [10]. At an organizational level, there is a need to adhere to local governance or NHS Trust guidance, which may fluctuate over time [11,12].

Although there is good-quality research outlining the use of various digital platforms for people with intellectual disabilities to support social contacts [13], there is little evidence about whether and how they use technology to manage their health. While the impact of video or telephone consultations (VCs) has been widely studied, their effects on specific patient groups, such as people with intellectual disabilities, remain less well understood [14].

In this paper, we use the term VCs, as it is favored by our study participants, community partners, patient and public involvement and engagement (PPIE) contributors, and the Experts by Experience (EbE) group. This term represents both telephone and video modes of consultation to assist any aspect of health care (eg, to support an annual health check), which complement in-person care delivery.

During COVID-19, people with intellectual disabilities, their families, and support workers (SWs) experienced a reduction and change in health care provision [8]. These compounded challenges encountered when accessing health care and exacerbated existing health inequalities experienced by people with intellectual disabilities [15,16]. Since COVID-19, the challenge has been to embed the use of technologies more widely despite having fewer resources and needing to work differently. With greater reliance on technologies to speak to HCPs, there is a need to explore the experience and views of people with intellectual disabilities who might struggle more with virtual service provision. While several UK policies highlight the need to improve uptake of key services for this group, such as annual health checks [2] and digital access [17], greater understanding is required about whether and how VC may widen opportunities for care access and integration across GP and community care providers.

The overall aim of the Experience-Based Co-Design (EBCD) study was (1) to explore the views and experiences of VC from the perspective of people with intellectual disabilities, their families, SWs, GP, and community care professionals and (2) to coproduce practical resources and guidelines to support the access and use of VCs. In this paper, we report findings for objective 1, users’ views and experiences of VC, as related to quality and safety. The development of the co-designed resources from objective 2 will be reported in a subsequent paper.

## Methods

### Early Project Design

Our study developed from early PPIE work, comprising a series of meetings over 24 months and a brief survey conducted in 2020. This accessible survey of people with intellectual disabilities explored how they talk to HCPs and their views of interacting via video or telephone calls. Findings from the meetings and survey helped shape and refine the aims of this study and informed the study design, ensuring that our methods were accessible and the purpose was meaningful to participant groups.

### Design

We adopted an EBCD research design [18]. This participatory and collaborative method promotes service-user involvement to drive health care quality improvement [19,20]. Using participatory, narrative, and visual methods, it provides a practical and collaborative way to engage people with intellectual disabilities [21]. This method also aligns with national guidance about how services should be planned and delivered, emphasizing coproduction with people with intellectual disabilities, their families, and SWs [22]. In keeping with inclusive research principles, we established an EbE group to work with us throughout the study to help inform the accessibility of participant information, advise on recruitment strategies, and support data analysis (among other key tasks) to meet study milestones. This group comprised 9 people with intellectual disabilities and/or family members of people with intellectual disabilities.

**Figure 1:**
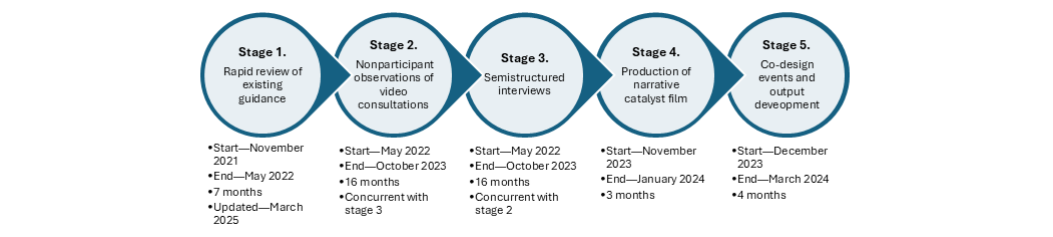
Time frame of each EBCD study method. EBCD: Experience-Based Co-Design.

The overall EBCD project comprised five sequential stages: (1) rapid review of existing guidance; (2) nonparticipant observation of video consultations in GP and community care; (3) qualitative semistructured interviews with people with intellectual disabilities, their family members or SWs, and GP and community care professionals; (4) production of narrative catalyst film, based on interview video clips; and (5) series of co-design and priority-setting events and meetings with participants. Stages 2 and 3 were undertaken concurrently. In this paper, we focus on data collected during observations (stage 2) and interviews (stage 3). The film production and co-design process and outputs will be reported elsewhere. Figure 1 shows the time frames associated with each stage.

### Sample and Recruitment

Participants included adults with intellectual disabilities, their family members or SWs, and HCPs recruited through GPs, community teams, and third-sector organizations across the Southeast of England. A purposeful maximum variation sampling strategy [23] was used to ensure participation from across a large geographical area (Kent, Surrey, Sussex, and South London) and diversity according to specific demographic variables, including gender, ethnicity, and locality. Eligibility criteria for people with intellectual disabilities included being ≥18 years and having a formal diagnosis or self-identifying as having mild to moderate intellectual disabilities. We extended eligibility criteria to also include family members or SWs for individuals with profound or severe intellectual disabilities due to their critical role in managing the person’s health. Participants could be supported throughout the study by a family member or SW. Participants needed internet access or a telephone (mobile or landline) connection. The involvement of family members and SWs was essential, particularly for participants with profound disabilities and those living in supported accommodation. Two people with intellectual disabilities consented to participate in the study but withdrew due to being unavailable for the interview.

Multiple recruitment strategies were used. For patients and families, recruitment was conducted via word of mouth, posters and flyers to our community intellectual disability partners, attendance and presentations at Intellectual Disabilities Communities of Practice meetings, and project notifications via their online or printed newsletters. Posters were also displayed in GP surgeries aimed at patients and their families or caregivers. Recruitment was also supported by word-of-mouth project introduction by HCPs in GP sites and via the research team’s professional network to promote the study. Audio and easy-read information were used throughout to support diverse recruitment.

HCPs were recruited via newsletters and support from primary care networks and clinical research networks. Presentations at community or local authority caregiver meetings were undertaken, followed by online meetings for professionals working in supported living or daycare facilities. Physical posters were also used in GP settings.

Eligible professionals were involved in the delivery or organization of health care services, including VC and annual health checks, within GP or community care teams. All participants resided or worked within Southeast England or South London, an area covering 4 integrated care boards or NHS trusts. Clinical sites’ withdrawal was due to misalignment of the recruitment period with work demands (COVID-19 and seasonal flu or pneumonia vaccine rollouts; 1 GP site), poor timing (1 supported living home), limited capacity to take part in research (1 community team), and reduced research and design (R&D) capacity combined with needing to maintain business continuity (1 community team).

### Data Collection

Data were collected from May 2022, with the entire project ending in 2024. All observations and interviews were conducted online using audio and visual recordings by an experienced social care researcher (TP). Adults with intellectual disabilities, their family members, and SWs were based either in their family home or a supported living home. HCPs participated from their workplace. Interview topic guides were informed by existing literature, our previous PPIE work, co-designed with our community partners, and piloted with our EbE group. We offered a flexible approach to project participation, with participants choosing to take part in 1 or more stages of data collection (ie, observation, interview, and co-design). Participants were unknown to the research team prior to the commencement of the study. Data were collected using Microsoft Teams, and all recordings were uploaded into a Trusted Research Environment [24,25].

### Ethical Considerations

Project information packs, comprising project information and consent forms, were available in various accessible formats, including easy-read, audio, and printed paper. Seven types of project information packs were developed to best suit our different participant groups (patients, family members, SWs, and HCPs) and to ensure that we addressed diverse participant groups’ accessibility needs. We obtained written and oral consent at each stage. Efforts undertaken to maintain participants’ privacy included anonymization of transcripts, removal of identifying information, secure storage, and access to data using a Trusted Research Environment secure server. We gained ethics approval from the NHS Health Research Authority (HRA reference: 303071) and the University of Surrey (reference: 2021/15 FHMS). Our EbE group and co-design participants were compensated for their contribution to the project, with interview and observation participants generously volunteering their time.

### Nonparticipant Observation

Two of the research team (FM and TP) members drove the observation recruitment. Once potential participants contacted us, we posted or emailed out the relevant project information pack. Once we received consent, we contacted the patient, family member or SW, or HCP to answer any questions and talk about the logistics of the nonparticipant observation. Questions tended to be about how the observation would work, the time taken, what would happen in the consultation, and what would happen at the end. Participants who were willing to proceed were then invited to a new meeting to test their laptop or phone connectivity and other device functions (camera, sound, etc) being used for the observation. This additional meeting was optional, but all participants chose to meet with us, as they found it reassuring ahead of the observation. The researcher (TP) was present at the start of the VC to introduce themselves and remind them that the VC would be recorded. The recording then started, and the researcher withdrew from the VC, leaving the HCP, patient, and SW to conduct the consultation. An observation schedule was developed and used after observation to ensure consistent recording of field notes (available on request). The schedule was informed by key concepts such as communication style, as outlined by Roter and Larson [26] and Miller and Nelson [27], and constructs from the Consolidated Framework for Implementation Research [28]. The schedule comprised 97 topic areas, which were evidenced as being important in health consultations, such as the consultation environment, technical support, consultation purpose or aim, communication, and patient-health care staff relationship. The researcher also made observations and reflection notes when reviewing consultation data. The goal of the observation was to observe real-world consultations to better understand the experiences of VC participants, gaining insights into the process by which VCs are planned, conducted, and followed up, and the interpersonal and relational impact. By viewing the VC, the researchers would also gain a better understanding of participants’ experiences, as reported in subsequent stages of the study.

Three observed consultations took place between HCPs and patients, with a family member or SW if preferred. Although we initially aimed to observe up to 12 consultations, practical constraints, such as extended recruitment and preparation timelines, led us to reassess our approach. Following 3 completed observations, we determined that sufficient data had been gathered to support meaningful interpretation. Consequently, we discontinued further recruitment for observation. In total, 10 participants were involved in these 3 consultations. Observations lasted between 20 and 75 minutes and were all video consultations.

Interviews (n=34) were supported by an interview schedule comprising 17 questions across 7 topic areas, focusing on the context of VCs, likes and dislikes of VCs, access and choice, relational and communication, safety and quality, workload and VC service fit, and finally, future use and advice for people thinking of using VCs. Table S1 in Multimedia Appendix 1 outlines the interview schedule topics and example questions. Interview schedules varied depending on whether the participant had taken part in the VC observation, used VCs before, or by their role (patient, family member, SW, or care professional). Postinterview field notes were completed to record any researcher observations and reflections. Interviews lasted 20-90 minutes. Two patients chose to be supported by an SW at the interview.

Our target interview number across geographical areas and participant groups was 36. Although we had arranged the remaining 2 interviews, these were not conducted, as interviewees became unresponsive after they requested to reschedule their interview date. Toward the end of the interview period, it became evident that no new themes or insights were emerging. Consequently, we determined that we had collected sufficient interview data to support robust and meaningful analysis.

### Data Analysis

Analysis was conducted in several stages. First, the data were analyzed separately according to the data collection method. Observational data comprising field notes and observation schedule data were tabulated to become familiar with the data across observations, followed by inductive and deductive coding. These codes were informed by key communication styles [26,27] and other frameworks [28] and brought together with the interview data at the indexing stage.

Framework analysis was used to analyze interview data, supported by NVivo (version 14; QSR International Pty Ltd). Framework analysis consists of 5 analytic stages (familiarization, identifying themes, indexing, charting, and mapping and interpretation) [29,30]. Interview data were transcribed verbatim, and inductive and deductive codes were attributed to segments of data, staying close to the context and participants’ meaning. We created codes based on the emerging data, but we were also mindful of key frameworks [26-28]. Two researchers (TP and FM) conducted independent coding, after which they met daily for 1 week to agree on codes, descriptors, and index codes, which shared commonalities [29,30]. Charting, where data were rearranged or clustered, to create order, was undertaken by the same researchers in a series of meetings before finalizing at 2 consensus meetings with the wider research team (AC, VT, JA, PS, and HM). These meetings were used to map and begin interpreting the data, focusing on quality and safety issues across different time points (before, during, and after a VC), and examining whether the data were concordant, discordant, or complementary. Over 219 codes were initially created, which were refined to 25 themes and subthemes.

### Data Integration and Theoretical Frameworks

Themes from observation and interview data were tabulated across participant groups (patient, family member or SW, and HCP) and at key time points in the VC (before, during, and after). The data triangulation method was used to identify recurring, complementary, contrasting, and important overarching or cross-cutting themes [23]. Integrated data were presented as a bubble figure, aiding data visualization when discussed and refining data interpretation by the wider research team meetings (FM, PS, AC, HM, JA, and VT). At this stage, we refined themes constructed from our interpretations of data in 3 online meetings with our EbE. Data integration identified several overarching themes emerging from the analysis. The first of which was quality and safety, as it was the primary concern reported by participants. The mapping and interpretation of quality and safety data were synthesized into 5 subthemes. The study is reported in accordance with the COREQ (Consolidated Criteria for Reporting Qualitative Research) framework) [31].

## Results

### Participant and Care Setting Characteristics

Three VC observations were undertaken, with 10 participants. A total of 34 interviews were conducted, and of these, 22 were with a range of HCPs in GP and community care settings (Table 1).

**Table 1:** General practice and community care professions and care setting (n=22).

Characteristics		Care setting				
		Primary care or general practice (n=5), n (%)		Community care teams (n=13), n (%)		Supported or care professional (n=4), n (%)
**Gender**						
	Men	—^a^	—		1 (25)	
	Women	5 (100)	13 (100)		3 (75)	
**Professions**						
	General practitioner	1 (20)	—		—	
	Practice nurse	1 (20)	—		—	
	Intellectual disability liaison nurse	1 (20)	—		—	
	Care coordinator	2 (40)	—		—	
	Intellectual disability community matron	—	1 (8)		—	
	Speech and language therapists	—	3 (23)		—	
	Specialist nurse	—	2 (15)		—	
	Physiotherapist or lead	—	3 (23)		—	
	Clinical psychologist	—	1 (8)		—	
	Clinical psychiatrist	—	1 (8)		—	
	Community care professionals (coordinators and managers)	—	2 (15)		—	
	Home managers	—	—		3 (75)	
	Care professional	—	—		1 (25)	
**Geographical**						
	Kent (n=7)	2 (40)	4 (31)		1 (25)	
	Surrey (n=8)	2 (40)	4 (31)		2 (50)	
	Sussex (n=5)	1 (20)	3 (23)		1 (25)	
	Out of area (n=2)	—	2 (15)		—	

^a^Not applicable.

Patient and family participants comprise people with mild to moderate intellectual disabilities (n=6), family members, including those of people with profound intellectual disabilities (n=2), and SWs (n=4; total n=12). Patient, family, and SW interview demographics are outlined in Table 2. Patients were equally split between men and women. SWs (n=4) worked in supported living homes (n=2) or day centers (n=2). All SWs were women. Family members were a parent of adult patients and a brother. Patients lived in a range of homes, independently in their own home (n=1), family home (n=2), and supported living homes (n=3).

**Table 2:** Patient or family and support worker interview characteristics (n=12).

Characteristics			Participant				
			People with intellectual disabilities (n=6)		Family members (n=2)		Support workers (n=4)
**Gender, n (%)**							
	Men	3 (50)		1 (50)		—^a^	
	Women	3 (50)		1 (50)		4 (100)	
Age range (years)			26-68		—		—
**Residence or work setting, n (%)**							
	Home (with family)	2 (33)		—		—	
	Home (independent)	1 (17)		—		—	
	Supported living home	3 (50)		—		2 (50)	
	Day care service	—		—		2 (50)	
**Relation, n (%)**							
	Parents of adults with profound intellectual disabilities	—		1 (50)		—	
	Brother	—		1 (50)		—	

^a^Not applicable.

### Summary of Overarching Themes

Overall, a mixed picture emerged from our analysis. There was agreement across all participant groups that the best practice care delivery for people with intellectual disabilities was in-person appointments. However, under certain conditions, VCs may complement existing health care services, particularly for patients who experience significant stress or discomfort during in-person appointments. As an HCP said, “I think it [VCs] will complement. I don’t think it can replace” (D027 HCP).

Five themes were identified, which encompass key considerations for conducting high-quality VC safely: (1) context, space, and purpose; (2) choice; (3) familiarity, online relationships, and trust; (4) prepare and personalize; and (5) continued connection. Key themes and the associated subthemes are shown in Table 3. Illustrative quotes are presented in Table S2 in Multimedia Appendix 1.

**Table 3:** Key themes and subthemes by participant group.

Participant group				Key insights
**Theme 1: context, space, and purpose**				
	**Subtheme 1: home and work context**			
		People with intellectual disabilities	Privacy to talk Safe and familiar physical space	
		Family members and support workers	Privacy to talk Agency and choice to leave or stay in the room VC^a^ as a tool to improve access and confidence	
		HCPs^b^ (GP^c^ and community)	Knowing who is in the room and privacy Missing vital signs Ability to control who is in the room and agency Appropriate workspaces	
	**Subtheme 2: urgency, pain, and purpose**			
		People with intellectual disabilities	Deciding urgency Purpose of the appointment	
		Family members and support workers	Assessing urgency, pain, and timeliness Considering travel and transportation	
		HCPs (GP and community)	Assessing urgency and pain Importance of timely access and clear purpose Diagnostic challenges (eg, overshadowing)	
	**Subtheme 3: physical checks**			
		People with intellectual disabilities	Medication review or understanding Seeking help when worried	
		Family members and support workers	Role ambiguity or drift and delegation Hybrid consultation, home equipment, and perceived risk	
		HCPs (GP and community)	Complement in-person care and limitations Hybrid consultation	
**Theme 2: choice**				
	People with intellectual disabilities		Support from family or support workers in practical tasks Facilitating comprehension and support Importance of choice and autonomy	
	Family members and support workers		Flexibility around patient and family preferences Confidence in using technology	
	HCPs (GP and community)		Responsiveness to patient preferences and service flexibility Confidence in technology use	
**Theme 3: familiarity, online relationships, and trust**				
	People with intellectual disabilities		Developing familiarity and unique communication styles Building trust	
	Family members and support workers		Building familiarity Building trust Confidence in what is being seen and heard Relationships with HCPs	
	HCPs (GP and community)		Building familiarity and individual communication Building trust Trust in what is being seen and heard Foundations for ongoing contact HCPs’ relationships with families	
**Theme 4: prepare and personalize**				
	People with intellectual disabilities		HCP preparedness, including awareness of reasonable adjustments Importance of HCPs’ understanding communication styles (verbal and nonverbal)	
	Family members and support workers		HCP preparedness, including frustrations over the lack of awareness of reasonable adjustments Importance of HCPs’ understanding varied communication styles (verbal and nonverbal)	
	HCPs (GP and community)		Presence of family members during VCs Need to establish contingency planning Value of collaborative learning within care teams	
**Theme 5: continued connection**				
	People with intellectual disabilities		Feeling connected Loss of connection and quality Avoiding interruptions Post-VC information in accessible formats	
	Family members and support workers		Ensuring continuity Connecting long-distance families or support workers Mixed experiences of connection and community Minimizing interruptions Helpfulness of post-VC information	
	HCPs (GP and community)		Ensuring continuity, including service ethos Importance of family members or support workers in maintaining connection Potential for fragmented care, the unintentional consequence of VCs Consulting space and interruptions Importance of post-VC information	

^a^VC: virtual consultation.

^b^HCP: health care professional.

^c^GP: general practice.

### Theme 1: Context, Space, and Purpose

Consideration of the patient’s context, such as their home environment and medical needs, and the purpose of the consultation, including whether any physical checks were required, was reported to be essential in determining the quality and safety of conducting a consultation virtually, rather than in-person. Context was seen as critically important at all stages of VC planning, delivery, and follow-up.

#### Home and Work Context

For HCPs, prior knowledge of the patient’s home context (ie, shared, independent, or family homes) and whether the person had the necessary support (eg, family, spouse, sibling, or SW) to use video or telephone devices was the first step for determining the appropriateness of a VC.

HCPs recognized the potential benefits of VCs, but they reported the need to continuously weigh these against possible risks. HCPs raised concerns about their limited awareness or control over who was in the virtual “room.”

... we’ve certainly had situations where we haven’t been clear, who’s been in the room when we’ve been doing virtual consultations and we’ve not been told that there’s like a whole bunch of other people sitting on the sofa listening. And that made us think like, Ohh, actually we need to specifically ask, like, who’s in the room because it just felt very odd to think that there were people that were listening but not contributing to a conversation.

E007 HCP interview

HCPs, therefore, encouraged everyone participating in a VC to introduce themselves (if unknown) and to be visible on screen. They were also concerned about missing vital signs or identifying risks, such as bad smells (urine), or client neglect (not dressed or unclear clothes). HCPs reported drawing on existing knowledge and prior experience of risk factors within the patient’s home context to inform consultation planning and decisions about the appropriateness of offering a VC.

People with intellectual disabilities, their families, and SWs emphasized the value of a VC occurring in a physical space, which facilitated feeling safe and a sense of privacy so they could talk openly. These home environments were often described as quiet and uncluttered spaces. However, HCPs questioned whether patients, or even themselves, had access to appropriate meeting spaces to meet virtually. HCPs reported sometimes working in dark, nonsoundproofed rooms, or workspaces that had unexplained issues, such as random, frequent alarms. They also worried about inadvertently screen-sharing other patients’ personal details, if using multiple screens, during the appointment, which could impact privacy.

Staff also reported factors related to the sense of agency when conducting VC with patients in their home setting. HCPs spoke about their limited ability to control who was present during consultations and which rooms the VCs were conducted in. Limited control over who was present also limited HCPs’ feelings that they could request parents of adults with intellectual disabilities to leave the room when necessary. HCPs also mentioned the “limited return” of VCs, observing that patients could easily walk away during consultations and choose not to return. Walking away was less common during in-person consultations. While HCP generally viewed the presence of family members or SWs during VCs as beneficial, they did note that in certain situations, such as when parents were present, it could hinder open discussions of sensitive topics, including sexual health with young adults.

For people with intellectual disabilities and their families or SWs, being able to leave a consultation was sometimes experienced positively, giving them a greater sense of agency and control. This raises questions about whether it is easier or harder to “leave the room” in an in-person versus a VC appointment and suggests whether contingency planning is needed, in case patients “opting out” of virtual appointments altogether. As such, the rules of engagement were more challenging to navigate online.

HCPs also questioned whether VCs empowered or disengaged patients, particularly when multiple participants attended, such as in multidisciplinary care planning meetings, or when rooms were unsuitable for VCs. For SWs, VC may offer patients greater autonomy, and with this comes the autonomy to disengage from appointments. In contrast, SWs reported feeling more confident in VCs, rather than in in-person appointments, when talking to clinical staff and other agency workers:

... it’s easier for me to sort of like bounce ideas and stuff rather than being in a room full of people where I’d be a bit, maybe a bit more quiet.

B015 SW interview

This was because they were in a familiar environment and were comfortable with working online. This suggests how VC can impact professional dynamics, with some professionals feeling more empowered to share their views virtually.

For people with intellectual disabilities, VCs were sometimes preferred, with family members reporting how VCs were beneficial when patients were too nervous to leave the home for in-person appointments. Family members also reported how VCs may appeal to the heterogeneity of people with intellectual disabilities across age groups, widening their potential appeal.

In summary, several key factors impacted the perceived safety of VCs conducted in the home, and these include having an appropriate quiet, usable virtual space, consideration about who would be in attendance, and all participants’ confidence (patients, families, SWs, and HCPs) when talking online or using technology.

#### Urgency, Pain, and Purpose

The timeliness and urgency of the appointment (urgent or nonurgent routine) were critical in assessing whether an in-person or virtual appointment was needed. Decisions were influenced by factors such as pain levels, type of service needed (ie, speech and language therapy and general practitioner), and travel logistics (eg, cost, distance, and mode of transport). Families or SWs played a crucial role in recognizing when someone was in pain, further shaping these decisions. HCPs recognized the importance of assessing urgency or pain levels to determine whether virtual or in-person care was required. In making these decisions, many HCPs highlighted diagnostic overshadowing, where symptoms can be misattributed to an existing diagnosis, such as an intellectual disability, leading to missed or delayed care [32-34]. Diagnostic overshadowing was a significant challenge, particularly in virtual settings, where identifying pain was more difficult.

They [carers] often say this thing of “they’ve got a high pain threshold” which all it means is that the adult can’t express their pain. They’re still feeling the pain, they’re just not able to express it. Whereas sometimes, if you’ll actually see the adult, you can see if they move differently or their guarding themselves, you can see they’re probably in pain, but you can’t really get that virtually all the time.

E006 HCP interview

Missing expressions of pain emerged as a primary concern for HCPs and SWs alike. Identifying the signs of pain depended on HCPs’ knowledge of the patient and their unique characteristics, health history, and how they express discomfort.

Confidence in decision-making relied on recognizing individual pain expressions and determining whether escalation was needed. In such circumstances, telephone and video appointments were reported to be more challenging, as greater emphasis was placed on HCPs’ ability to assess what is seen or unseen in virtual appointments. None of the HCP participants mentioned using established tools to identify pain [32].

Although VCs were seen as more challenging to conduct for HCPs, they offered advantages in delivering timely care. While physical checks required in-person visits, the urgency of appointment decisions was weighed against appointment availability, travel feasibility, and pain levels. Further risk considerations arose when patients were known to underreport key symptoms. In this situation, HCPs, families, and SWs highlighted the need to be familiar with the patient to assess urgency and for HCPs to offer prompt appointments when needed. Prior knowledge of a person’s typical responses was key in distinguishing visible and subtle signs of pain. This was considered by HCPs as central to identifying pain or discomfort and/or signs of neglect or abuse. Being familiar with the patient helped, especially if SWs or HCPs were concerned about the patient underreporting pain to avoid treatment or in-person care.

Having a clear purpose for the VC was important for people with intellectual disabilities. In this study, we identified several practical uses of VC, including pre- and postcare information gathering, medication checks, imparting nonurgent test results, and care planning meetings before and after annual health checks. VCs were also used to support different types of appointments, including those with speech and language therapy, physiotherapy, and neurology. HCPs and SWs reported VC as being particularly useful for people with a history of nonattendance and preexisting conditions, including those who are housebound, have phobias, are immunocompromised, and/or have challenging behavior. Some of these issues were also relevant for family members, with one noting that being immunocompromised themselves made in-person attendance a less favorable option. People with intellectual disabilities took part in VCs for a range of activities, such as a medication review or follow-up on a new treatment. HCPs and SWs also worked together to collate physical measurement (ie, weight) data when in-person appointments were considered too stressful for patients or when travel or transport barriers made attendance difficult.

In summary, assessing urgency and identifying pain were the main factors influencing HCPs’ and SWs’ views of VC. These factors were important in understanding the quality of VCs. Assessing urgency requires being familiar with the patient and knowing their signs of distress. For people with intellectual disabilities, having a clear understanding of the VC’s purpose was paramount, preferring in-person appointments if the conversation was sensitive, serious, or complex.

#### Physical Checks

There was some agreement between all participant groups about how and when VC could be used to support physical checks. As noted earlier, acceptance of use was initially based on the topic under discussion. Mental and sexual health topics were generally considered unsuitable for VCs, as these were seen as potentially triggering, unsafe, or inappropriate when talking to patients, especially when families or SWs were present. HCPs reported specific activities that were successfully undertaken online, such as information gathering before annual health checks, medication reviews, or as a stand-alone VC to follow up on nonurgent test results, check medication or equipment delivery or use, or update on next steps in care planning. HCPs also used VC to prepare for in-person appointments, but only when needed.

There were mixed views about VC use to support activities such as medication review. While most HCPs favored VC to support medication reviews, people with intellectual disabilities often found phone consultations challenging, particularly due to difficulties in understanding information clearly.

Sometimes I can’t understand, the pharmacist [on the telephone], they’ve got a pharmacist there, which I don’t understand .... With the doctor it’s OK, because I understood. But with the pharmacist querying the medication, I couldn’t. Don’t know. I couldn’t hear him properly ....

D025 patient interview

People with intellectual disabilities spoke about needing prior knowledge about the topic to be discussed in the VC before deciding whether an in-person or a VC was best. For patients, in-person appointments were preferred if a physical check was needed or if they were worried about something.

In relation to physical checks, VCs were reported to sometimes blur boundaries between the roles of SWs, HCPs, and family members. For example, a researcher noted in one observation how a practice nurse asked a female SW if a breast examination had previously been undertaken. When it had not, the nurse then asked if the SW could assist the patient in performing the examination. The SW appeared reluctant, as the patient was known to be uncomfortable with being touched. The researcher’s observation notes recorded the SW’s facial expressions, showing discomfort and frowning to show a lack of agreement with the request. The researcher noted that the SW’s reaction was likely because such an activity fell outside their typical responsibilities. Similarly, the HCP went on to ask the SW if they were able to take vital signs, like a blood pressure (BP) reading. This potential drift of delegation might have significant implications for the quality and safety of health care, as reliance on SW’s skills or knowledge and willingness to perform these tasks becomes paramount. This drift also has the potential to blur professional and quality boundaries, especially for SW who might not feel comfortable or informed about taking vital or assisting in health-related tasks.

Several strategies were adopted when physical monitoring was needed, but in-person attendance was not possible due to stress or other impediments. In these situations, a hybrid consultation style was observed with HCP asking SW or family members to undertake and report readings such as body weight and BP.

HCPs and family members considered a hybrid style of care delivery useful, but in very defined situations. Hybrid working relies on SWs or families accurately recording data and ensuring that readings are reported back to HCPs. This reliance was reciprocal, as HCPs also relied on families or SWs to help with essential care when options for in-person appointments were limited (eg, when communicating vital sign information, care, or medication instructions). The challenges of monitoring vital signs (ie, BP, urine, and temperature) also emerged in interviews with families. They were concerned about HCPs’ expectations that families had the skills and equipment at home to perform physical readings.

Overall, despite the obvious limitations of using VC, HCPs and families also described some benefits of this hybrid approach to care, but these were dependent on several factors, such as whether the appointment covered low-risk or routine topics. They also spoke about their potential benefits in supporting more frequent attendance, over that of just annual health checks. This suggests that VCs may play a role in widening access opportunities for patients, enabling patients to consult more frequently and complementing in-person care.

### Theme 2: Choice

During interviews, people with intellectual disabilities reported that they sometimes required support from a family member or SW. Partners, parents, or siblings often assumed practical responsibilities, such as taking notes or providing supplementary information throughout the entire VC process: before, during, and after the consultation. They also assisted with communication by rewording, rephrasing, or clarifying information to reduce the risk of misunderstandings and ensure that the individual’s verbal or nonverbal communication was accurately understood. This also included support with comprehension and information retention—including explaining instructions, note-taking, and arranging reminders: “Information, my key worker or my parents will explain things to me” (D026 patient interview).

Patients spoke of how having someone with them facilitated comprehension, before, during, and after appointments. Having appropriate support helped allay worries:

It means that you can have you can be calm and relaxed and you can have the people you need around you. Like in my case where I’ve got my other half and my brother, I can have them both here and they can hear exactly what’s being said.

D002 patient interview

For people with intellectual disabilities, feeling comfortable was also linked to practical considerations, such as knowing the purpose of the appointment, who will be there, such as a trusted advocate, and knowing what, if any, activities will take place. Other practical considerations were knowing how long the appointment would be. The need for support was particularly important when planning and undertaking the first VC, as previous negative experiences influenced attitudes toward future VCs for almost all groups.

Challenges related to ensuring privacy during VCs were evident for HCPs when both patients and their family members were present on the call. In some cases, individuals wished to speak privately with the HCPs but were unable to do so within the constraints of the virtual format. However, similar privacy concerns were also reported during in-person appointments, particularly when family members were accompanied by younger children due to the unavailability of childcare.

Knowing and prioritizing patients’ and family members’ preferences and choices for the mode of consultation emerged as a quality indicator. Patients and family members valued HCPs who prioritized their preferences, whether for an in-person appointment or a VC, and were acutely aware of when VCs were used in ways that appeared to prioritize the service needs over their own preferences. This tended to occur when services had limited staff availability, time, staff transportation issues, or their home was geographically inaccessible.

Being flexible about offering choice and taking account of preferences, and how these may change over time, was acknowledged by HCPs as an important consideration when building familiarity and rapport. Participants with intellectual disabilities frequently emphasized the importance of having choice in how care was delivered, while also acknowledging that preferences might vary depending on the specific health concern or the need for physical examinations. This flexibility also included consideration of staff preferences, about how they chose to work, and if they themselves had reasonable adjustments or disabilities that impacted on delivering care virtually (ie, having sensory impairments).

Finally, supporting choice, in practical terms, may depend on patients, families or SWs, and HCPs’ confidence in using technology and their ability to organize, set up, and open VC appointment links. Many patients owned or had access to tablets, smartphones, and laptops and were confident in using them to communicate online through different platforms:

I’m used to the iPad cause I did loads of things on my iPad. I use zoom. I use Microsoft. And to adapt the way we are now.

D026 patient interview

For patients in supported living, they frequently had access to laptops owned by the home manager. As previously noted, family members and SWs played a central role in supporting communication and comprehension during VCs. Unlike in-person appointments, though, families or SWs also needed to have some technological competency to effectively support communication in the virtual environment.

Offering patients a choice in the mode of contact (in-person, video, or telephone) was recognized as an indicator of high-quality care. However, this is contingent upon HCPs’ ongoing assessment of patients’ evolving needs and preferences. Facilitating choice also requires consideration of various contextual factors, including the role of families and SWs in supporting VC, the availability of appropriate digital devices, and the digital literacy of users.

### Theme 3: Familiarity, Online Relationships, and Trust

Building familiarity was seen as essential to building good online relationships. Once established, a good rapport contributed to multiple forms of trust, including trust in HCPs’ competence in using VC, their ability to deliver meaningful consultations, trust in the technology itself, and confidence that care would remain continuous and that choice in care delivery would be maintained. Building familiarity was seen as knowing the patient, their health status and care plans, family context, support network, and ideally, their interests, hobbies, or jobs. Building familiarity was the foundation for the first contact and successful ongoing engagement:

I mean the thing more that helped because I’d already met her face to face. So I knew who I was talking to, so it might have been more nervous if I hadn’t known her. It was that I was going to have on the screen.

D002 patient interview

HCPs reported that building familiarity was the focus in the first VC, before any subsequent meetings focusing on clinical issues. This first VC was especially important for those reluctant to attend in-person appointments. Sometimes an in-person appointment was needed first, before moving online, when regular ongoing care was needed, and there were concerns that regular in-person attendance would not be possible. Developing familiarity with HCPs was perceived by all participants as contributing to more positive future interactions, whether through virtual or in-person appointments. Building familiarity also aided feelings of comfort and was a key part of laying the foundations of future care. For both patients and HCPs, this sense of familiarity helped foster trust, reduce anxiety, and improve communication through better rapport, all of which are critical to enabling person-centered care. Linked to familiarity, all participant groups mentioned the pros and cons of communicating online and the need to build familiarity to help understand individuals’ communication styles, unique characteristics, and sense of humor. Indeed, HCPs often noted their surprise at the extent to which meaningful relationships could be established online, even in cases where they had not previously met the patient, family, or SW in person:

You know, we’re building up a relationship with them and and it’s surprised me how well that worked for people that haven’t met us in person. And then when we would go out and see them in person ... they would know who we were because they’d seen us on the screen ....

E004 HCP interview

Familiarity between VC participants also extended to knowing, not just the patient, but also the SW, supported living or day care staff and/or home managers by name, as their roles were essential in initiating VC, building rapport between professional groups, and maintaining ongoing contact.

Trust in the use of a VC was also shaped by what families or SWs and HCPs could observe and hear during video or telephone appointments. A limited or partial view of the patient, such as only seeing them from the waist up, was a concern for HCPs, as it restricted their ability to assess the patients holistically. However, the impact of this limitation varied across professional roles, with some specialist staff adapting their use of VC to suit their clinical needs. For instance, a community physiotherapist repositioning the camera to capture the patient walking around the room.

For HCPs, having advanced communication skills was considered key to establishing a trusted relationship to ensure VCs went well. HCPs reported using multiple skills aimed at getting to know the patient, such as taking time for chit-chat, authentically asking questions about clients’ interests, and advanced skills needed to build rapport (tone, pace, eye contact, and body language). The absence of this authentic contact could otherwise reduce HCP to be, “... just a head in a box” (E011 HCP interview).

Building familiarity and relationships with families or SWs was important for HCPs, as VC contributed to efficient care planning over time. The flexibility of using VC also allowed for the inclusion of family members and other care agencies (eg, social care professionals) in appointments, regardless of their physical location. This was particularly beneficial in accommodating family members who worked shifts or had other scheduling constraints, enabling broader participation in patient care.

Overall, there were mixed views about the ease of building online relationships and the many factors needed to establish and support trusted relationships to develop over time.

### Theme 4: Prepare and Personalize

Preparing for VCs was considered critical at different time points, before, during, and after a VC, as the tasks varied according to the context and purpose of the appointment. Examples included sending preappointment reminders or post-VC information checks. Only patients, family members, or SWs spoke about the importance of HCPs being prepared for online or telephone appointments. They emphasized the need for HCPs to be familiar with the patients’ needs, such as existing health conditions, reasonable adjustments, communication or support needs, and whether a patient would be accompanied by a family member or SW. Nevertheless, patients and families reported that this preparation was not often undertaken and sometimes overlooked entirely: “I think have health professions are so overworked that they don’t read the notes” (D002 patient interview).

Family members expressed frustration with HCPs’ lack of preparation for VCs, particularly when required to repeat essential information such as reasonable adjustments or communication needs, which were already documented in the electronic medical record or when such records or notes were not reviewed in advance.

HCPs occasionally questioned the presence of family members on the call. Recurring advice from people with intellectual disabilities for HCPs was to get to know the patient, as this would aid their understanding of the patient and improve the quality of the conversations once in the virtual “room.” Patients and families emphasized the importance of HCPs understanding their communication styles. Preparation was considered essential for enhancing both verbal and nonverbal communication, as the more prepared HCPs were for the VC, the more comfortable patients and families felt engaging with them.

Inevitably, technical issues arose during VC. HCPs emphasized the importance of early preparation, including establishing contingency plans at the outset of the consultation and, where possible, reinforced through prior correspondence. This plan typically involved informing patients that, in the event of internet connectivity failure, the clinician would immediately initiate a telephone call to continue the appointment. The presence of such an alternative arrangement provided reassurance to patients and reflected the thoroughness of clinicians’ preparatory practices for VCs.

Strategies to enhance VC use among HCP teams took several forms. For instance, HCPs routinely documented consultation experiences, briefed team members, and established procedures for consistently communicating summary notes or follow-up actions to patients and their families when appropriate. This collective learning aimed not only to build professional competencies and support the exchange of effective practices but also to mitigate the recurrence of poor practice. Ultimately, these efforts aimed to strengthen best practices and ensure the sustained quality of VC.

Overall, the success of VCs was dependent on key preparatory tasks carried out at different time points, including contingency planning by HCPs, awareness of reasonable adjustments, and the implementation of team-based improvement strategies.

### Theme 5: Continued Connection

For individuals with intellectual disabilities, repeated contact with the same HCP was especially valued, as it promoted continuity and reduced the cognitive and emotional demands associated with engaging with staff, particularly those mediated by technology. Patients liked and expected to see the same professional when they visited their GP or community team in person. This was similarly echoed in VC. Feeling connected to HCPs and services emerged in several ways throughout the study.

Continued connection was an important theme, before, during, and after a VC, and was seen as a quality indicator. HCPs reported their intention to plan future appointments for patients with the same staff member. This was seen as important not only to monitor health through regular checks but also to keep the momentum of being seen regularly and build on prior relationships and familiarity. For HCPs, continuity was embedded into the ethos of some services, with some explicitly stating that supporting continuity was one of their core values.

I just liaise with them and work around what’s best for them, who they want to see if they want continuity, if there’s a particular clinician they want to see or if there’s a site because we’ve got four sites they might want to only go to a particular site. So I’m always on the phone speaking to them.

D028 HCP interview

VCs could facilitate ongoing engagement in care, particularly for family members and SWs who wished to be involved in decision-making but were unable to attend appointments in person due to geographic distance or other commitments. VCs offered a flexible means of participation, enabling individuals to join consultations partially or fully, thus maintaining involvement in the care process. This continued connection was perceived as a valuable opportunity to share information more efficiently and to respond more promptly to emerging needs in care planning and management. For patients, VCs may serve as an initial point of contact with HCPs, allowing for the development of rapport even before any in-person appointments, again helping to reinforce the sense of connection through familiarity with HCPs.

Although VCs may facilitate connection with health care services, family members, SWs, and HCPs expressed concerns that VCs could also lead to a loss of connection or a perception of impersonal care, describing them as more “clinical”:

I feel like having the kind of virtual side of it kind of depersonalises it again and the whole part of my work is personalisation and, kind of, making every kind of contact count. I don’t know if necessarily sometimes video chats is the best way forward for it really.

E003 CP interview

For patients, families or SWs, and HCPs, feeling connected was enhanced if VCs were uninterrupted, such as when HCPs put patients on hold or were disturbed by colleagues asking questions. These interruptions were thought to impact the flow of conversation and level of engagement, making a difference to whether patients felt comfortable or not. For HCPs, being interrupted was strongly linked to whether they had an appropriate (quiet) meeting space or had all the information they needed prior to starting the appointment.

The conclusion of a VC was regarded as a critical phase for ensuring continuity of care. Health care staff emphasized the importance of clearly communicating the next steps, including any required follow-up, aftercare, or planned activities. Explicit articulation of postconsultation actions ensured that all participants were informed about subsequent processes, which helped to manage expectations and facilitated a smooth transition to ongoing care. This included sending post-VC written summary notes, preferably in accessible formats, using plain language, easy-read, and different communication modes (letters and telephone calls).

All user groups expressed the importance of continuity of care, specifically the value of meeting the same HCP during VCs. Feelings of disconnection were alleviated by simple measures such as ensuring that calls were uninterrupted. A sustained sense of connectedness was perceived as integral to the quality provision over time.

## Discussion

### Principal Findings

In this paper, we report the first UK-based insights into VC and the factors influencing the quality and safety of use for individuals with intellectual disabilities, their families, SWs, and HCPs. Our findings highlight vital areas for development to inform how services are designed and delivered in the future and enable sustainable, safe, individualized, and effective care [35,36], contributing to a deeper understanding of VCs as a form of online service provision for all user groups. This learning can be used to underpin how services are designed and delivered in the future, enabling safe, high-quality, person-centered care. We found a complex perspective on the acceptability, use, and effectiveness of VCs in managing health care for people with intellectual disabilities [37].

Our study reports mixed views about the acceptability and use of VC in relation to quality and safety as defined in UK policy [38,39]. Quality and safety are crucial aspects outlined in the UK Health and Care Act 2022 [38] and the NHS Outcomes Framework 2014 [39], which impact 3 key aspects of care: clinical effectiveness, safety, and patient experience. As implementation of the NHS 10-year plan [2] advances, it is vital to ensure that digital services are of high quality and safe. Although not reporting on clinical effectiveness, our data identify vital and interconnected elements needed to ensure effective service delivery. Namely, the VC space, online relationship building, personalization, and continued connection were critically important areas that were reported as being key to ensuring the quality and safety of care provision.

More broadly, our findings align with concerns about VC use among patients with sensory, neurological, and musculoskeletal impairments [7]. Similar issues have been noted in other groups at risk of digital exclusion, such as older adults in rural areas [40] and those with limited language proficiency [41-43]. Building on the work of Halas et al [35], who explored the balance between virtual and in-person care, we found that the risks associated with VCs may be heightened for individuals with intellectual disabilities, potentially compromising care quality [44]. While our data reflect some of these concerns, it also aligns with other research, indicating that there are opportunities for positive online experiences [45], especially when supported or implemented effectively. If we are to improve access to care, such as annual health checks, more needs to be done to facilitate access to alternative routes to care [46].

### Comparison to Previous Research

Safety was ingrained across many of the key themes we identified, particularly linked to the spaces where VCs take place, both at home and at work. Consistent with previous research, both the patients’ home environment [47] and the HCPs’ virtual workspace played a significant role in shaping the quality of VCs. The importance of the VC consultation space has been highlighted in previous studies involving service users from marginalized groups, such as asylum seekers, and supported living residents [43].

In line with other studies, we found uncertainty about privacy within home or work settings for VCs, as it was reported to inhibit engagement, especially for HCPs who might not have suitable workspaces [48] or feel less confident working online [10]. Risk was also heightened if HCPs were in any doubt about the patient’s home environment [42]. Clinicians also reported mixed views about the use of VCs, especially when linked to practical tasks, such as when a physical examination [49] was required, or there were concerns about their ability to identify pain or care urgency. Consistent with Halas et al [35], we found instances where HCPs needed to balance risks when managing patients virtually. These skills rely on advanced communication abilities whereby HCPs must depend on what is visible, heard, and said on screen [50]. People with intellectual disabilities often prefer video calls to telephone consultations, primarily because they enable visibility of facial expressions and improve communication [51]. We found that although telephone calls retained value in low-risk contexts or for quick information exchange, they could be less effective, particularly if affected by accents or a lack of visual cues. Nevertheless, like in-person care, HCPs need specific skills such as “empathetic verbalization” to enhance active listening [51]. Moreover, it could be argued that communication skills are even more important in VC, as there is a greater risk of missing vital information.

We found that patient experience was heavily linked to the development of trust through building good relationships, preparing for appointments and personalizing care, and critically ensuring continued connection, again to ensure uptake of future appointments. HCPs were vocal about the need to be familiar with the patient, which, in turn, built trust and paved the way for future appointments, whether in person or via VCs. Similar to other studies, we identified how prior relationships can support a reverse shift, from online interactions to in-person appointments, as these interactions had built a foundation of trust [52]. As such, VC may create new opportunities for therapeutic connections, especially when trust has already been established [37].

In our study, VC participants were aware of how the use of technology may have the potential to impact the patient-HCP relationship [35,44,53]. HCPs were observed to use multiple strategies to build familiarity and mitigate or eliminate the power imbalance that could occur in VCs. Building familiarity was based on recognizing VC participants’ unique communication styles and their ability to pick up on visual cues that aided understanding during appointments. Like other studies, our study found that body language and facial cues were key in supporting communication [50]. Our study also showed a close link between familiar and established relationships and communication effectiveness [54] due to families’ or caregivers’ ability to recognize the needs of patients and their role in supporting technology use. VCs offered opportunities for patients who may struggle with verbal communication in telephone or in-person appointments to access care in a way that made them feel more comfortable, such as patients with autism spectrum disorders. Consistent with prior research on remote care access for marginalized groups [55], VC can lead to different care experiences depending on how they are planned, participants’ familiarity with one another, and the context of their work or home environments.

VC requires preparation for planning, conducting, and undertaking post-VC tasks, and this can be a lengthy process. Previous studies have also emphasized the importance of HCP preparedness [56] and the need to balance access with workload demands [57]. Preparation takes time and includes a range of tasks over time to ensure choice is given, the VC is prepared for, and there is a continued connection. If any of these issues are absent, then there is a danger of future nonattendance and reduced opportunities for future VC engagement. As such, preparation is critical if care delivery is to be safe and of high quality. Similar to other studies, we found that a lack of HCP preparedness often led to patient and family frustration, especially if reasonable adjustments were not read before appointments or acted upon [58,59].

Finally, we identified that feeling connected and having a sense of continuity of care in VC was important for patients and their families. Continuity, especially seeing the same HCP, was linked to higher-quality care through improved rapport and familiarity. This aligns with existing evidence on sources of resilience [60], confirming the importance of connectedness, even in virtual spaces, and the concept of “accumulated knowledge” that underpins person-centered care [61]. Continuity may be even more important when consulting online, as the bond established can be argued to be more fragile than in-person appointments, especially if patients are unfamiliar with the HCP and trust has not been established.

### Implications for Practice, Education, and Research

Our findings point to implications for practice in terms of supplying HCPs with adequately equipped VC workspaces, IT equipment and support, and private or quiet workspaces. There is also a need to develop guidelines to support rules of engagement [62] or “netiquette” guidelines for online interaction [8,50,53]. Guidance on the rules of engagement, rapport, and relationship building online may also impact HCPs’ and SWs’ confidence in using VC and have implications for improved interaction. These considerations are all within the context of VC as a complement to service delivery, rather than a replacement for in-person care.

Not all HCPs were comfortable with video or telephone conferencing, but all wanted to improve their digital or communication skills. This highlights the need for education, training, and skill development at all levels of higher education and service types. HCPs suggested shared training opportunities with colleagues from related services to enhance IT skills and build confidence in conducting quality VC. It is essential that training is collaboratively designed by a diverse range of staff and includes digital support for all user groups, particularly for individuals with intellectual disabilities and family members or caregivers who are less confident IT users who may struggle with accessing digital health care [63].

Further research is needed to design user-friendly digital platforms that avoid excessive information and complex language [64,65]. Understanding how to foster successful online relationships over time is also crucial for both HCPs in different health care settings (HCPs) and individuals with intellectual disabilities [52,66]. Studies should also examine how VCs impact pain identification, waiting times, and care quality and satisfaction [67]. While some research addresses diagnostic overshadowing [33,68], for individuals with intellectual disabilities, knowledge about how technology-mediated health care affects timely access and health outcomes remains limited. Future studies should involve people with intellectual disabilities through co-design processes [69] to create inclusive technologies, focusing on areas such as cyber etiquette [70], online safety [71,72], and collaborative learning to establish best practices.

This study provides valuable insights into the use of VC across care settings. However, the transferability of the findings should be considered, as more data were derived from community teams rather than from GP professionals. Nonetheless, key findings, such as the importance of relationship-building, preparation, and continuity of care, are likely to be transferable and relevant across diverse settings and professional groups.

Future research could examine how these findings translate into other settings with differing technologies, information systems, or staffing models to better understand more subtle differences and the applicability of VC care delivery. Moreover, greater research into the possible ethical or medical-legal implications of professional role blurring may also be important, not only for health care employers to protect staff, but also more broadly in terms of providing ethical quality care.

For patients and families, key safety concerns arise when using VC, particularly in shared home settings and when there is a lack of practical support and guidance. Outputs from this study, including guidance, go some way to addressing these needs and may offer valuable insights for local authorities, health services, and policymakers to ultimately support the inclusive and responsible use of VC, complementing in-person provision. See Multimedia Appendix 1 for the web link to resources.

Finally, our project highlights several policy implications across GP, community care, and SW groups. In alignment with other research on digital inclusion [73], our research shows the need to align working practices across care settings to ensure consistent delivery of VC practices, ensuring appropriate workplace fit, and being attentive to supporting access and use of virtual care. Moreover, changes to current policies and best practice guidance are needed to overcome professional, service, and system-level barriers. Clarifying delegated tasks, between HCP and families or SWs, when taking vital signs, may also help define role boundaries between groups.

There is also a need to clarify when and how to use VC effectively. This should be supported by adequate resourcing in improved workspaces, targeted HCP or SW training, and co-designed resources for users. Additionally, VC platform design must improve to enhance digital accessibility, including visual and easy-read navigation tools and information for diverse user groups.

### Limitations

On reflection, one limitation was the number of observations undertaken. Initially, we aimed to observe up to 12 videoconferences. However, due to the challenges of arranging these sessions and time constraints within the project, we were only able to observe 3, 2 of which were in GP. A significant factor affecting recruitment was the pandemic itself, as most of our data collection took place during the latter end of this challenging period. Furthermore, although we captured a broad representation of community care professionals across geographical areas, our final sample lacked sufficient diversity of family members, especially those caring for people with profound intellectual disabilities and primary care clinicians, which may have caused sampling bias.

We gained valuable methodological insights from conducting this study, some of which highlighted real-world issues impacting research practices (both positive and negative, which will be reported elsewhere). As such, a further limitation might be that several interviews took place in unconventional but private spaces, such as home hallways or remote buildings not usually used for the service, as these were the only locations where family members, SWs, or HCPs could be interviewed and where internet connections were available or reliable.

These limitations were mitigated by the study’s co-design approach, focusing on quality and not speed. In this study, we collaborated with individuals with intellectual disabilities, their families, SWs, and community partners using co-design methods, all of which took time. This limitation was also this study’s strength, as this inclusive research approach was designed to ensure the collection of meaningful data that addresses the project’s goals and produces outputs that are useful, practical, and relevant in real-world settings.

### Conclusions

This study provides a deeper understanding of VCs as a form of online service provision. The findings reveal mixed opinions regarding the acceptability and use of VCs among individuals with intellectual disabilities, their families, SWs, and HCPs. Our results show how VCs can be conducted safely while ensuring high-quality, person-centered care. However, quality and safety can be compromised if not implemented effectively. We found that while VCs can play a significant role in delivering timely care, participants felt that they should not be considered a substitute for in-person consultations, nor should they be conducted in isolation. Instead, VCs can effectively complement in-person care, supporting existing care arrangements and even wider care access in specific circumstances. They also provide an opportunity for those who may not be able to attend in-person meetings to connect with HCPs in a way that meets their needs.

## Data Availability

The datasets generated or analyzed during this study are not publicly available due to controlled access arrangements but are available from the corresponding author on reasonable request. Extracted data are supplied in Multimedia Appendix 1, available to download along with the published manuscript.

## Appendix

Multimedia Appendix 1 Quotes and resources weblink.

## Abbreviations

BP: blood pressure
COREQ: Consolidated Criteria for Reporting Qualitative Research
EBCD: Experience-Based Co-Design
EbE: Experts by Experience
GP: general practice
HCP: health care professional
NHS: National Health Service
PPIE: patient and public involvement and engagement
SW: support worker
VCs: virtual consultations

## References

[1] Song K, Hey M, Payne R (2024). Media depictions of primary care teleconsultation safety: a thematic analysis of UK newspapers. Br J Gen Pract.

[2] (2025). 10 year health plan for England: fit for the future. Department of Health and Social Care.

[3] (2023). Digitally enabled triage. NHS England.

[4] (2023). Online health and prescription services. NHS Services.

[5] Payne R, MacIver E, Clarke A (2024). Do new models of primary care risk exacerbating existing inequity?. Br J Gen Pract.

[6] Patient safety healthcare inequalities reduction framework. NHS England.

[7] Pettersson L, Johansson S, Demmelmaier I, Gustavsson C (2023). Disability digital divide: survey of accessibility of eHealth services as perceived by people with and without impairment. BMC Public Health.

[8] Chadwick D, Ågren KA, Caton S, Chiner E, Danker J, Gómez‐Puerta M, et al (2022). Digital inclusion and participation of people with intellectual disabilities during COVID-19: a rapid review and international bricolage. J Policy Pract Intellect Disabil.

[9] Clarke G, Dias A, Wolters A (2022). Access to and delivery of general practice services: a study of patients at practices using digital and online tools. The Health Foundation.

[10] Statton S, Jones R, Thomas M, North T, Endacott R, Frost A, et al (2016). Professional learning needs in using video calls identified through workshops. BMC Med Educ.

[11] (2024). Digital requirements guidance. NHS England.

[12] (2023). Digital primary care: the good practice guidelines for GP electronic patient records—(GPGv5). NHS England.

[13] Flynn S, Hatton C (2021). Health and social care access for adults with learning disabilities across the UK during the COVID-19 pandemic in 2020. Tizard Learn Disabil Rev.

[14] Selick A, Durbin J, Salonia C, Volpe T, Orr E, Hermans H, et al (2022). The nuts and bolts of health care: evaluating an initiative to build direct support professional capacity to support the health care of individuals with intellectual disabilities. J Appl Res Intellect Disabil.

[15] Health and care of people with LD, experimental statistics 2022-2023. NHS England.

[16] White A, Sheehan R, Ditzel N, Ding J, Roberts C, Magill N, Yu MKL, Keagan-Bull R (2023). LeDeR Annual Report: Learning from lives and deaths people with a learning disability and autistic people. The Institute of Psychiatry, Psychology and Neuroscience (IoPPN), King's College London.

[17] (2024). Priorities and operational planning guidance 2024/25. NHS England.

[18] Bate S, Robert G (2007). Bringing User Experience to Health Care Improvement: The Concepts, Methods and Practices of Experience-Based Design.

[19] Tsianakas V, Robert G, Maben J, Richardson A, Dale C, Griffin M, et al (2012). Implementing patient-centred cancer care: using experience-based co-design to improve patient experience in breast and lung cancer services. Support Care Cancer.

[20] Tsianakas V, Robert G, Richardson A, Verity R, Oakley C, Murrells T, et al (2015). Enhancing the experience of carers in the chemotherapy outpatient setting: an exploratory randomised controlled trial to test impact, acceptability and feasibility of a complex intervention co-designed by carers and staff. Support Care Cancer.

[21] Kaley A, Hatton C, Milligan C (2019). More than words: the use of video in ethnographic research with people with intellectual disabilities. Qual Health Res.

[22] (2018). Learning disabilities and behaviour that challenges: service design and delivery. Nice Guideline Ref NG93. National Institute for Health and Care Excellence (NICE).

[23] Patton MQ (2015). Qualitative Research Methods: Integrating Theory and Practice.

[24] Dunford M (2025). Unlocking data secrets safely with Trusted Research Environments. Lifebit.

[25] UK Health Data Research Alliance, NHSX (2021). Building Trusted Research Environments—principles and best practices; towards TRE ecosystems. Zenodo.

[26] Roter D, Larson S (2002). The roter interaction analysis system (RIAS): utility and flexibility for analysis of medical interactions. Patient Educ Couns.

[27] Miller EA, Nelson E (2005). Modifying the roter interaction analysis system to study provider-patient communication in telemedicine: promises, pitfalls, insights, and recommendations. Telemed J E Health.

[28] Damschroder LJ, Aron DC, Keith RE, Kirsh SR, Alexander JA, Lowery JC (2009). Fostering implementation of health services research findings into practice: a consolidated framework for advancing implementation science. Implement Sci.

[29] Ritchie J, Spencer L, Bryman A, Burgess B (1994). Qualitative data analysis for applied policy research. Analyzing Qualitative Data.

[30] Gale NK, Heath G, Cameron E, Rashid S, Redwood S (2013). Using the framework method for the analysis of qualitative data in multi-disciplinary health research. BMC Med Res Methodol.

[31] Tong A, Sainsbury P, Craig J (2007). Consolidated criteria for reporting qualitative research (COREQ): a 32-item checklist for interviews and focus groups. Int J Qual Health Care.

[32] Clinical guide for frontline staff to support the management of patients with a learning disability and autistic people—relevant to all clinical specialties. NHS England.

[33] Mason J, Scior K (2004). ‘Diagnostic overshadowing’ amongst clinicians working with people with intellectual disabilities in the UK. J Appl Res Intellect Disabil.

[34] Dell'Armo K, Tassé MJ (2024). Diagnostic overshadowing of psychological disorders in people with intellectual disability: a systematic review. Am J Intellect Dev Disabil.

[35] Halas G, Baldwin A, LaBine L, MacKay K, Singer A, Katz A (2024). A phenomenological inquiry of the shift to virtual care delivery: insights from front-line primary care providers. Healthcare (Basel).

[36] Hammersley V, Donaghy E, Parker R, McNeilly H, Atherton H, Bikker A, et al (2019). Comparing the content and quality of video, telephone, and face-to-face consultations: a non-randomised, quasi-experimental, exploratory study in UK primary care. Br J Gen Pract.

[37] Lake J, Jachyra P, Volpe T, Lunsky Y, Magnacca C, Marcinkiewicz A, et al (2021). The wellbeing and mental health care experiences of adults with intellectual and developmental disabilities during COVID-19. J Ment Health Res Intellect Disabil.

[38] UK Parliament (2022). Health and Care Act 2022. Legislation.Gov.UK.

[39] (2015). NHS Outcomes Framework 2015 to 2016. Department of Health and Social Care.

[40] Landgren S, Cajander Å (2021). Non-use of digital health consultations among Swedish elderly living in the countryside. Front Public Health.

[41] (2021). Locked out: digitally excluded people's experiences of remote GP appointments. Healthwatch.

[42] Kaihlanen A, Virtanen L, Buchert U, Safarov N, Valkonen P, Hietapakka L, et al (2022). Towards digital health equity—a qualitative study of the challenges experienced by vulnerable groups in using digital health services in the COVID-19 era. BMC Health Serv Res.

[43] Moschogianis SF, Darley S, Coulson T, Peek N, Cheraghi-Sohi S, Brown B (2024). Patient experiences of an online consultation system: a qualitative study in English primary care post-COVID-19. Br J Gen Pract.

[44] Selick A, Bobbette N, Lunsky Y, Hamdani Y, Rayner J, Durbin J (2021). Virtual health care for adult patients with intellectual and developmental disabilities: a scoping review. Disabil Health J.

[45] Caton S, Bradshaw J, Gillooly A, Hatton C, Flynn S, Oloidi E, et al (2022). Digital participation of people with profound and multiple learning disabilities during the Covid‐19 pandemic in the UK. Br J Learn Disabil.

[46] Owens J, Ravindrarajah R, Norman G, Hopkin E, Shi C, Lovell K, et al (2025). Access, inequalities and annual health checks (AHCs) for adults living with severe mental illness in the UK: a mixed-methods systematic review. BMJ Open.

[47] Sachdeva N, Tuikka A, Kimppa K, Suomi R (2015). Digital disability divide in information society: a framework based on a structured literature review. J Inf Commun Ethics Soc.

[48] Turner A, Morris R, Rakhra D, Stevenson F, McDonagh L, Hamilton F, et al (2022). Unintended consequences of online consultations: a qualitative study in UK primary care. Br J Gen Pract.

[49] Lunsky Y, Bobbette N, Selick A, Jiwaet MI (2021). “The doctor will see you now”: direct support professionals’ perspectives on supporting adults with intellectual and developmental disabilities accessing health care during COVID-19. Disabil Health J.

[50] Selick A, Durbin J, Hamdani Y, Rayner J, Lunsky Y (2023). "Can you hear me now?": a qualitative exploration of communication quality in virtual primary care encounters for patients with intellectual and developmental disabilities. BMC Prim Care.

[51] Vennik J, Hughes S, Lyness E, McDermott C, Smith KA, Steele M, et al (2023). Patient perceptions of empathy in primary care telephone consultations: a mixed methods study. Patient Educ Couns.

[52] Scheffers F, van Vugt E, Moonen X (2020). Resilience in the face of adversity in adults with an intellectual disability: a literature review. J Appl Res Intellect Disabil.

[53] Desroches ML, Stych J, Bannett G, Guttentag R, Ailey SH, Fisher K (2024). Establishing best practices in telehealth care for adults with developmental disabilities in the United States: an e-Delphi study. Telemed J E Health.

[54] Menschik C, Kunze C, Renner G, Etges T (2024). Mainstream technologies in facilities for people with intellectual disabilities: multiple-methods study using the nonadoption, abandonment, scale-up, spread, and sustainability framework. JMIR Rehabil Assist Technol.

[55] Humphrey A, Cummins S, May C, Stevenson F (2025). GP remote consultations with marginalised patients and the importance of place during care: a qualitative study of the role of place in GP consultations. BJGP Open.

[56] Boardman L, Bernal J, Hollins S (2018). Communicating with people with intellectual disabilities: a guide for general psychiatrists. Adv Psychiatr Treat.

[57] Neve G, Fyfe M, Hayhoe B, Kumar S (2020). Digital health in primary care: risks and recommendations. Br J Gen Pract.

[58] Accessible information standard. NHS England.

[59] (2023). DAPB4019: Reasonable Adjustment Digital Flag. NHS England.

[60] Scheffers F, Moonen X, van Vugt E (2021). Assessing the quality of support and discovering sources of resilience during COVID-19 measures in people with intellectual disabilities by professional carers. Res Dev Disabil.

[61] Hjortdahl P (1992). Continuity of care: general practitioners' knowledge about, and sense of responsibility toward their patients. Fam Pract.

[62] Mold F, Cooke D, Ip A, Roy P, Denton S, Armes J (2021). COVID-19 and beyond: virtual consultations in primary care-reflecting on the evidence base for implementation and ensuring reach: commentary article. BMJ Health Care Inform.

[63] Chadwick D, Wesson C, Fullwood C (2013). Internet access by people with intellectual disabilities: inequalities and opportunities. Future Internet.

[64] Cooper‐Moss N, Umpleby K, Roberts C, Garner C, Edwards AH, Ditzel N, et al (2024). Barriers to healthcare for people with a learning disability from ethnic minorities: perspectives of self‐advocates and carers. Brit J Learn Disabil.

[65] Savolainen K, Kujala S (2024). Testing two online symptom checkers with vulnerable groups: usability study to improve cognitive accessibility of eHealth services. JMIR Hum Factors.

[66] (2025). Reasonable Adjustment Flag. NHS England.

[67] Pereira Gray DJ, Sidaway-Lee K, White E, Thorne A, Evans PH (2018). Continuity of care with doctors-a matter of life and death? A systematic review of continuity of care and mortality. BMJ Open.

[68] Jopp DA, Keys CB (2001). Diagnostic overshadowing reviewed and reconsidered. Am J Ment Retard.

[69] Dakin FH, Rybczynska-Bunt S, Rosen R, Clarke A, Greenhalgh T (2024). Access and triage in contemporary general practice: a novel theory of digital candidacy. Soc Sci Med.

[70] Caton S, Chapman M (2016). The use of social media and people with intellectual disability: a systematic review and thematic analysis. J Intellect Dev Disabil.

[71] Triantafyllopoulou P, Clark-Hughes C, Langdon PE (2022). Social media and cyber-bullying in autistic adults. J Autism Dev Disord.

[72] Triantafyllopoulou P, Newsome J, Tsang W, McCarthy M, Jones K (2025). Safer online lives: internet use and online experiences of adults with intellectual disabilities-a survey study. J Appl Res Intellect Disabil.

[73] Alami H, Lehoux P, Shaw SE, Papoutsi C, Rybczynska-Bunt S, Fortin J (2022). Virtual care and the inverse care law: implications for policy, practice, research, public and patients. Int J Environ Res Public Health.

