# Supplementary material for: Virtual Consultations for People With Intellectual Disabilities in General Practice and Community Care: Mixed Methods Qualitative Study
Source: J Med Internet Res. 2026 May 20;28:e81173. doi: 10.2196/81173 (PMC13234538; doi:10.2196/81173)
Supplement: Multimedia Appendix 1 [file jmir_v28i1e81173_app1.docx]

**Table S1. Interview schedule - Topics and questions**

|  | Topic | Example Questions |
| --- | --- | --- |
| 1. | Context/usual care | - Please can you tell me briefly about your service and role? |
|  |  | - How does your service use, or would like to use VCs? |
| 2. | Likes & dislikes of VC | - What do/would you like about using VC for your patient group? |
|  |  | - If used before, were there any problems? What were these? How were these issues resolved? |
| 3. | Experiences, access & preferences | - What could have helped your experience? |
|  |  | - How prepared/confident were/are you at conducting a VC? |
|  |  | - In thinking about your role, was there anything that could have helped you to improve the VC experience? |
| 4. | Relational & communication | - Were/would there be any topics you preferred not to talk about using VC? Why? When would you not use a VC? |
|  |  | - Were there any issues with communicating or providing information in the VC? |
|  |  | - Would/have you have any concerns about asking questions? If not, why not? |
| 5. | Safety, quality, risk | - Do/have you any concerns about any aspect of conducting a VC? What are these concerns/worries? What could reduce these worries? |
|  |  | - Were there any unexpected benefits from using VCs? |
| 6. | Workload & fit | - How are VCs integrated into current care? How do they currently “fit” with existing work practices? Was there any impact on workload? |
|  |  | - What kinds of changes do you think would be needed to make VC work better in your work setting/ for people with intellectual disabilities? |
| 7. | Advice and future use | - Would you use a VC again? Why? Why not? |
|  |  | - What advice would you give to other professionals who are thinking about offering VCs for people with intellectual disabilities? - Finally, what kind of training, information or support would be useful to you/your service when providing care via VC? |

**Table S2. Patient, Family/Support Worker and Healthcare Professional Interview Quotes**

| **People with Intellectual Disabilities (PwID)** | **Family Members (FM) & Support Workers (SW)** | **General Practice & Community Healthcare Professionals (HCP)** |
| --- | --- | --- |
| **Theme 1. Context, Space and Purpose** | | |
| **Home Context** | | |
| ***Physical space which facilitates a feeling of safety and privacy to talk***  “I can't sit in now talking to people. At the moment it's quiet, but if I do sometimes, I can hear like banging. I can hear people outside. And it really affects me, it affects me. I can't and I need it to be quiet.” D024 Patient interview  “The dining area was fairly busy and there was some noise from people talking in the room. Also possible TV or radio noise in the background. Unseen. Other PwID and staff regularly walked behind the camera and on one occasion one person waved at the laptop. Other staff members occasionally joined in the conversation to help answer queries they knew the answers to”. Observation 1 Fieldnotes  “It seemed a relaxed environment but busy. The noise was commented on by the HCP and on one occasion the PwID turned to see what was going on.” Observation 1 Fieldnotes  “It seemed a relaxed environment but busy. The noise was commented on by the HCP and on one occasion the PwLD turned to see what was going on.”  (1 Fieldnotes) | ***Privacy to talk***  “I think the only risk would be if, obviously if there was people listening in to conversation. I mean, you want to keep things as private and personal as possible… But I think if it was more personal, things like doctor's appointments, you wouldn't want to be, you'd have to go somewhere private. You wouldn't want everyone listening in to all their thing. I mean, we're all very careful about that, you know, if someone's got a problem, we always take them off. We don't, wouldn't stand in a room full of people and discuss anything like that. ” B015 SW interview  “…talking about issues which might be seen as sensitive (bowels etc) in an open assisted living space”. Observation 2  Fieldnotes  “I still think it's that one to one consultation, when, when, when the doctor can see, you can see them, you're able to be able to. There are markers and indications that you can't necessarily see like this (indicating over a video call) that you can when you're in a one-to-one situation in my opinion.” A003 FM PwPID interview  ***Agency and choice about whether to leave a room***  “I remember once we were trying to do somebody's, they have like a PCP meeting (person-centred planning). So they have anybody who supports them in a meeting and them and the lady didn't have Wi-Fi or any devices and we had them in the office in her armchair, which was really difficult (laughing). With her core team, myself, the other manager and it was horrendous because the office, is really tiny and she got really, really fed up. So in the end, she just chose to leave the meeting. It was crammed and she was fed up. It was boring for her.” B013 SW interview  ***VC potential to widen access to care***  “I mean there might, there might be some that would prefer who are neurodivergent because they're so anxious and nervous about leaving their homes that a virtual consultation would actually be more applicable. And because I know they’re neurodivergent these young people that I support, they might actually prefer that.” A003 FM PwPID interview  ***Opportunities for SW to feel more confident***  “Being able to to chat. And I think it's easier for me to sort of like bounce ideas and stuff rather than being in a room full of people where I'd be a bit, maybe a bit more quiet. And when we used to have the team briefings while we were still at home during lockdown, felt like I would say a lot more on the screen rather than if I was, everyone was in the same room, I probably wouldn't. I'd leave it and then speak to my manager afterwards. So yeah, I definitely prefer it and you can talk to people more.” B015 SW interview. | ***Missing vital signs***  “I think for me it's all the stuff you miss out from going to visit someone. You can't see the environment properly, you can't. Smells are really important in an assessment, you know. Sort of how people are interacting together is very difficult to see properly online.” E011 HCP interview  “… when you go into an environment…sometimes you go in and there'll be a strong smell of urine and you’ll know that's not quite right, but you also miss all that interaction stuff. I think. … when you're doing an assessment, you're using every part of your senses. What you can see, you can hear, you can smell for an assessment and that's really, really important.” E011 HCP interview  ***Privacy to talk***  “The dining area was fairly busy and there was some noise from people talking in the room. Also, possible TV or radio noise in the background. Other supported living residents with learning disabilities and staff regularly walked behind the camera/laptop and on one occasion one person waved at the laptop.” Obervation 1 Fieldnotes  “…there was a lack of privacy because of all the people around, and the fact that other staff members were able to join in at various times without invitation. Observation 1 Fieldnotes  ***Appropriate workspaces***  “...in my building we have this bizarre alarm that goes off every sort of 25 minutes and it just goes up and it really freaks out my clients with sensory needs. E008 HCP Interview  “I guess just my unusual circumstances of wheelchair user. So there are residential services in that area that aren't accessible to me. And so I am able to meet people I guess in their home environment remotely that I wouldn't be able to in person.” E010 HCP interview  “And so you know a lot of people living in Group homes may not have access to a private space that would feel comfortable and safe for them to engage on a virtual call…” E007 HCP interview  “Trying to get a room is an issue sometimes.” A001 HCP interview  ***Weighing the circumstances and possible risks of a virtual visit***  “And then being able to be in their home environment, that feels safe and calm and you know good and positive for them. But it means that they can access the therapy easier. So again, there are advantages on that side, but it feels like there's a lot of clinical judgement in working out where you might kind of push for one or another.” E007 HCP interview  ***Knowing who is in the room***  “… not being necessarily told by the person on the screen, who else's present has certainly posted some challenges in the past, whether that's other family members who just randomly chip in halfway through or, you know, people with a learning disability who you know might not wanna sit and listen to their parents telling you all their different ways that they exhibit challenging behaviour for an hour and you wouldn't put them through that if you knew that they were there. … ” E007 HCP interview  ***Ability to control who is in the room***  “I can't police that through a screen” E008 HCP interview  “I mean it's really hard because in the clinic, if I get the sense that somebody needs to tell me something that they don't want their mum to hear or they don't want their care to hear, I can physically say, right, OK, do do. It's all right if I just talk to you, I just, I'm going to ask you to step out. Now when it's talking to a thing [indicating a screen via Attend Anywhere (AA)], all I know is that I've asked them to step out. I don't know if they've gone around to the back of the laptop and they're still standing there and giving them the eyes and be like, “don't you say anything to that doctor”. E008 HCP interview  ***Agency & and choice about whether to leave a room/walk away***  “I'm aware of is there were some people with a learning disability and autism, who liked it because you weren't encroaching on their space and they felt they could get out of the situation. They were obviously people who were able to to express that. Not everybody we work with can.” E011 HCP interview  “And again, like if you had somebody in the room who got up and walked out, you would be fairly clear about the fact they'd made a choice there. You might follow them to find if they were OK, but you, you know what their choice has been. Whereas sometimes you might be in a situation where you've got someone who's quite distressed and then they disappear and you don't know what's going on there and you don't want to leave them if actually it was just a computer connection issue.” E007 HCP interview  “It's so much harder for people to assert themselves when there's just a row of faces in an MDT meeting on teams or Skype.” E008 HCP interview |
| **People with Intellectual Disabilities** | **Family Members & Support Workers** | **General Practice & Community Healthcare Professionals** |
| **Urgency, Pain and Purpose** | | |
| ***Purpose of the appointment and deciding urgency***  “If it's urgent, you’re (unclear word) doing that. But it's not urgent. Down the phone or the iPad.” D026 Patient interview  “Over the call, I wouldn't talked about cancer. I rather talk about that face to face. Private stuff. It should be. It shouldn't be over the phone. It should be face to face, but anything else is OK.” D024 Patient interview  ***Purpose of VC appointment***  “Well, one would be meds review because its easier [in-person].” D023 Patient interview  “I I think if you if the person needs to see you or it's something that they need to be able to explain to you face to face, you're worried about something, then I think you need the face to face. But if it's like she was just doing a general check in how you doing? How's your medication? As long as you've got enough understanding or someone with you that can say, well, this is this medication, I think you'd be OK with her.” D002 Patient interview | ***Assessing urgency***  “If I needed a doctor's opinion quickly or a physio's quickly any anything I needed quickly, I'll definitely consider virtual. If it was something that could wait, I'd rather wait for a personal appointment.” B013 Support Worker interview  “…there are times when, I think you know a phone call or being talked to over the phone. All like this is useful. Because the circumstances dictate that that it is, that it has to be done that way.” A003 FM PwPID interview  **“**Well, I mean, if there was anything like a physical examination needed, I suppose. I mean, it's very easy for someone still go over the phone. So yeah, I've injured myself. How bad the situation would actually be…” D002 FM interview  ***Timeliness***  “Ohh, it depends with the circumstances. Like for example, at the moment (name of PwID) she is very poorly so yesterday I just had to. I explained to the surgery her symptoms and we didn't have to go there and they managed to prescribe her antibiotics which she started today. So it will depend on situation to be honest. “ D001 SW interview  ***Travel and transportation***  “…it reduces the difficulty in terms of if someone can't travel.” B010 HM interview  “I had one patient and they wanted to have conversation about their medication. Um, so they had a quick chat with her GP via kind of video chat. Um, and then they were able to discuss a bit more about the symptoms and the side effects of the medication, which then encouraged them to come into the surgery to kind of have the blood test and have further appointments. Yeah.” E003 CP interview | ***Assessing urgency***  “And occasionally there'll be people who need to be seen urgently, but I can't get to them or they can't get to me and so I might have a quick kind of a quick interview via Attend Anywhere, but that's that is now the limit of it for me. … I've not. Not really enjoyed it and I think. I think there's a lot that you miss when you don't do face-to-face consults.” E008 HCP interview  ***Diagnostic overshadowing/ Assessing pain***  “Like some people with a learning disability, they don't present as having pain, but they can be an excruciating pain. But unless you see them, and you see is what their body's doing, how their bodies react in what the temperature is, what their eyes look like, what their respiration looks like and all that sort of thing.” D022 HCP interview  ***What is seen and unseen/prior knowledge***  “If they can't answer the phone, it might be an agency member of staff who doesn't know them well enough, doesn't know how they display pain. And it might be someone who knows them really well, but the GP feels they know better.” D022 HCP interview  “We're not a big advocate for them [VCs] as the the gold standard, as far as we're concerned and our Commissioners concerned is face to face for people with learning disabilities and because of the way they express pain, they present, you get diagnostic overshadowing a lot, which is where they see the person's behaviours as being attributed to their learning disability and not probably the physical health issue that they're struggling with.” D022 HCP interview  ***Timeliness***  “Um, I would say is sometimes what they're describing is not actually what's happening. So, they might say describe it in a certain way, say pain or itching. But then when you have a look at it, it's a rash or something. So having photos, having cameras, sometimes very helpful because by the time we come and see you, it could have actually disappeared. So, so that was helpful.” D027 HCP interview  ***Travel and transportation***  “I'm working quite a rural area so transport links aren't always very good or services maybe are short-staffed or don't have a driver on or whatever it is.” E007 HCP interview  ***Purpose of VC appointment***  “You know, you might find someone who they just want the medication reviewed or you get the frequent attenders who just want to talk to someone. Virtual consultations are very appropriate for those individuals as long as there's nothing alarming going on.” D022 HCP interview |
| **People with Intellectual Disabilities** | **Family Members & Support Workers** | **General Practice & Community Healthcare Professionals** |
| **Physical Checks** | | |
| ***Medication review/Understanding***  “Sometimes I can't understand, the pharmacist [on the telephone], they’ve got a pharmacist there, which I don't understand…. With the doctor it’s OK, because I understood. But with the pharmacist querying the medication, I couldn't. Don't know. I couldn't hear him properly …”. D025 Patient interview  ***If worried about something***  “I I think if you if the person needs to see you or it's something that they need to be able to explain to you face to face, you're worried about something, then I think you need the face to face.” D002 Patient interview | ***Role drift and Delegation***  “The nurse discussed with the team whether the support workers could gather info and send it to the clinic (check weight, BP etc. The SW said,  “…can’t imagine that would be possible” Observation 2 Fieldnotes.  ***Hybrid consultation, home equipment and perceived risk***  “I suppose it's about what it is that you're trying to, the outcome we're trying to achieve at the end... So if the outcome at the end is something that isn't necessarily going to mean further, doesn’t require further intervention then maybe a virtual consultation is the way forward. But if, say for example, we're doing the virtual annual health check and the boys have got really low, low blood pressure, how are they going to find that out by doing it over the phone? Unless they’re expecting us to have a blood pressure monitor here at home.” A003 FM interview | ***Hybrid consultations***  “I did sort of quite kind of physical and clinical sort of virtuals, the one was with the gentleman with The Walking aid and I think we were doing it via WhatsApp call and essentially I was giving instruction to his carer, who was then instructing him sort of face to face. And because that was a bit that was it was just too difficult to give instruction whilst to him from, you know, essentially I was in the room with him on a phone. So I think that it was done by his, you know, by his supporter who was next to him” E009 HCP interview.  ***Hybrid consultations***  “…one was with the …walking aid and I think we were doing it via WhatsApp call and essentially I was giving instructions to his carer, who was then instructing him sort of face to face…” E009 HCP interview.  **Complement in-person care and limitations.**  “I think it will grow in use because you can use it for certain specific patient groups, I think it can be complemented to the normal face-to-face consultation as well because as part of annual review per se, you might have additional checkups or to discuss results after that and it's a it's a way to talk with the patient and support worker directly without the without any problem. So I think it's a good tool and can be implemented especially in the Learning disability group people with Ohh great result for both parts actually.” D001 HCP interview  “… there's so much that still can't be done virtually. …what we do is go and train up HCAs [Health Care Assistants] and practice nurses to be able to do part of it [taking vitals]. And then the GP can come in for the last 10 minutes, do a physical health, to look over, to see if there's any rashes to worry about or any lumps in the groin for like hernias. And then the HCA or practice nurse can have done their blood pressure, weight, height, dipped their urine, and done bloods. And that's all the really vital part of the annual health check. So I don't know how that would be done virtually.” D022 HCP interview***.*** |
| **People with Intellectual Disabilities** | **Family Members & Support Workers** | **General Practice & Community Healthcare Professionals** |
| **Theme 2. Support choice** | | |
| ***Family/SW support in practical tasks***  “It means that you can have you can be calm and relaxed and you can have the people you need around you. Like in my case where I've got my other half and my brother, I can have them both here and they can hear exactly what's being said.” D002 Patient interview  ***Facilitating comprehension & support***  “And I find it hard to understand so. And I repeat myself sometimes”. and “I repeat myself. A lot of people don’t. And they use strong/easy words here and there ….“ D026 Patient interview  “I like a second person to be with me. …I forget things quite easily. And it's getting worse. So I have (support) there to take notes down if necessary.” D025 Patient Interview  When asked what would help, she responded “Oh yeah, by talking about, with my support worker or my parents… Talking before and afterwards.” D026 Patient interview  ***Own note taking to aid memory***  “Yes, I was kind of like jotting notes down as we were going through the review.” D023 Patient interview  **Allay worries**  “... how long the appointment, the telephone conference was for" D023 Patient interview.  “I suffer from depression and anxiety and depression and also I'm stated to be got autism as well. So it's to have someone with me at least then I know. I know that I won't get stressed or anything because they will calm me down.” D024 Patient interview  “Oh yeah, by talking about, with my support worker or my parents… Talking before and afterwards.” D026 Patient interview.  ***Importance of choice***  “I’d say as long as the person feels comfortable with it and their understanding is good enough that you know they're not gonna get anxious about it then. But I think it should be the person who it's about should make the final decision. Because at the end it's their, it’s their personal information. It's their decision. I, I think they can't really make it on their behalf. I know it's a lot harder when people look after people that are more, need more support.” D002 Patient interview  “It goes back to basically what support they need and what works for them as a person.”  D024 Patient Co-Design Event 1  “I think we all sort of came to the same point in the end the IT was really a choice and some people prefer to do it face to face. Some people like to have the option” D024 Patient Co-Design Event 1  “I've been, I like face to face sometimes. I like a phone call sometimes.” D026 Patient interview  “…it was just harder because it wasn’t face to face. I like speaking to somebody face to face not like, when it’s an appointment. It’s easier to get, your feelings across.” D023 Patient interview  ***Confidence in using technology***  “I used my tablet and then because it was connected to his Wi-Fi, so as long as the internet is OK, and the link works ok then it's fine. But I've done teams and that before through other things, so I knew what I was doing.” D002 Patient interview  [Researcher] And is there anybody that can help you with the tech problems? “I do it myself, I'm really good that I'm good at computers anyway.” and “Yeah, I've got tablet and mobile. It means I can have on my phone, on the tablet I can have zoom and on tablet and on phone I can have zoom, and I’m easier, I can talk to them.” D024 Patient interview  “I use a, I’ve got a tablet at home, and I’ve got a mobile phone as well. Researcher: Ok, so you’re quite used to technology then? Yes, that’s correct.” D023 Patient interview | ***Patient and family members' preferences and being flexible***  “I'd much prefer to just talk face to face to somebody then I'm not seeing me talking to you and I and I'm far more animated and I'm far more articulate when I'm not having to look at me (laughing).” A003 FM PwPID interview  “I think if you know as a choice for people, I think it's really got its benefits and its uses and and if it can. … can speed things up like you know, like … I, can I just do a quick …WhatsApp video you know? … that could mean like instead … three weeks for, you know, speech and language …you know, be able to find a slot to come and see her then, then that could you know, that could be a real sort of benefit.” E005 HM interview  “I'd say to just discuss it with your doctors or your health worker or whoever it is you'll be doing with first.” D002 FM interview.  “I think definitely offer it as an option to people rather than make it the accepted norm.” D029 CP interview.  “I think that'll always be the time where you'll need to be in the same room at someone. And I say, I don't think we should never lose that.” D002 FM interview  ***Confidence in using technology***  “Oh yes, I was like [at first] I can't do that. I can’t do that, but now I'm quite good on a computer (laughing).” B014 CP interview | ***Family members’ presence in VCs***  “And thinking of the remote consultation, … there was a young man, learning disability, he was also autistic and he was there with his mother and she was clearly wanting to say things, whilst he was present it was a hybrid consultation, so when I don't know whether that made a difference, but so one of my colleagues was in in the clinic room and I was sort of here. But Mum was wanting to say things. For my information and for my nursing colleagues information, but didn't want her son to hear. But that was very difficult to manage remotely like I I was still was struggling to follow quite what was going on.” E010 HCP interview  “I think the quality of the interactions very much dependent on who supports them and how they’re enabled or empower to take part and I think for some people that's worked well on the screen, yeah, that's definitely the biggest thing.” E004 HCP interview  ***Family presence at in-person appointments/Privacy***  “Whereas at home kids could be running, partner could be around, mother could be here. So it does limit a bit, in that sense.” D027 HCP interview  ***Taking account of preferences and service flexibility***  “Well, I suppose, I suppose my biggest question is, you know, if we are a service user-led service, what do they want? Is this actually in their best interest? Is this actually benefiting them? You know is it is it that it benefits us?” A002 HCP interview  ***Taking accont of preferences and service flexibility***  “Some people are still expressing a real preference for face-to-face, and that's what we're doing. So I guess, you know, in part, we're determined by the client preference, because if you know the repport is gonna be built better with somebody if they're clearly saying to you “I don't wanna have to sit in a room with people”, then, of course, they're gonna feel more relaxed and at ease online. So in that situation, I think the rapport was built better with that person online.” E007 HCP interview  “Well, I suppose, I suppose my biggest question is, you know, if we are a service user-led service, what do they want? Is this actually in their best interest? Is this actually benefiting them? You know is it is it that it benefits us?” A002 HCP interview  “I think definitely offer it as an option to people rather than make it the accepted norm.” D029 CP interview.  ***Supporting choice has implications for ways of working***  “And then really … getting the right support from within your services so that you've got the right IT support and the right operational and management support to be able to implement it. And then making sure that you've got, yeah, the staff working with the staff too because it within staff teams as well, different people have, you know, are kind of are more inclined to use it, or less inclined depending on the personal preferences of staff as well.” E009 HPC interview  ***Confidence in using technology***  “…to be honest, you know, I'm a mature student, so a technical issues are not are not my forte” A001 HCP interview |
| **People with Intellectual Disabilities** | **Family Members & Support Workers** | **General Practice & Community Healthcare Professionals** |
| **Theme 3. Building familiarity, online relationships and trust** | | |
| ***Building familiarity***  “I think if you had an appointment, someone you've never met, it's gonna make you more anxious.” D002 Patient interview  Researcher, “What did you like about the video call with HCP?” “Get on with her” Researcher: “And was it OK talking to her on the video call?” “Yeah.” B010 Patient interview  “The one I had that was I have for myself that one was quite hard because it was a doctor I'd never met before. So I didn't know who it was. Who was gonna be on the screen. And just had to deal with it on my own. Because unfortunately, my brother was busy at the time. And that made me quite anxious cause I was like I had a name and that was kind of it.” D002 Patient interview  “I think if you had an appointment, someone you've never met, it's gonna make you more anxious. You know, and I what to expect.” D002 Patient interview  “I lost my GP. She had left the practise after 17 years. And, and she was a lovely doctor. And she would take her time, with you, it wasn't a rush job. She was lovely. You know, she was late for always late for appointments, but I know why. Because she's always got this extra mile, you know she, she was a lovely doctor and I miss her.” D025 Patient interview  ***Unique communication styles***  “…we just had a normal chit chat and she's got my sense of humour. So she knew when I was sort of joking that she was putting me, she knew I wasn't being offensive. But it was my way of dealing with it.” D002 Patient interview  ***Building Trust***  “And trust. Trust is very important, to trust people.” D026 Patient interview  “..and the background knowledge [helps]...” D026 IO Patient interview | ***Building familiarity***  “…like I said, it was with our doctors. So there was that there was that level of comfort ready there. We, like I said, I I obviously think it's more, if it is with someone you know that's sort of helps a little bit.” D002 FM interview  ***Trust in what is beeing seen and heard.***  “I suppose if I could describe the symptoms to the consultant over the phone or the doctor over the phone in respect of the boys. That might be helpful, but it's more about the fact that they need to understand that I have to talk on their behalf without them getting all shirty with me and trying to, because the’re adults, trying to talk to them.” A003 FM interview  “That's the dilemma and it's when they it's every time like when we actually take the boys into a place. And I say, look, the boys are like this the boys are like that and it's almost like you're disbelieved. And then when they meet them everything changes. Yeah, that I'm telling the truth”. A003 FM PwPID interview  ***Building Trust***  “I do think that that's what rolls around the back of all of their minds. It's it's, you know. Is mum saying these things because mum's, you know, a Nutter and she wants to over medicate her children? Or is it because she’s really, genuinely concerned, has concerns about them and wants the best for them?” A003 FM PwPID interview  ***Families relationships with HCP***  “Now the brother is unable to attend it physically. It's only 5 minutes up the road for me from (residential home name) but he lives in London and he's quite an elderly gentleman, so, he doesn't drive, so it would be quite difficult for him to get down to the meeting so he'll be attending via teams. So in that aspect it's fantastic because we're still able to go ahead with the meeting, and the next of kin will have a very important impact on that meeting, you know.” BO10 HM interview  “I mean, so generally speaking with appointments, I have to base everything around my job. So it was easier in that respect because then I could just go straight to work afterwards. If I had a shift that day.” D002 FM interview | ***Building familiarity***  “I have a patient till now who refuses to come to the surgery, does not engage with any other services. She's housebound, she's in her 20s and lives with her mum. I've tried home visit once, that was four or five years ago. … we did a virtual one using video accurex video consultation. And it was, it was OK … she was able to communicate. … It was helpful because she never comes …So it worked well. …that was helpful in that situation. So, I think there are certain … groups of people that it might work, but there are limitations” D027 HCP interview  “In Ob 1&3 HCP both mentioned when/how they had previously met the patient.” Integrated Observation fieldnotes  “There were a lack of introductions in Ob 2, but in Ob 1 and 3 the HCP starts chatting with very open questions, inquiring about their [pts] day and how they were.” Integrated Observation Fieldnotes  ***Laying foundations for ongoing contact***  “But then you can always easily review people virtually. You know, once you've already met that client and you kind of know, OK, with this work for this person, then you can possibly do it virtually then, if that makes sense.” B010 HCP interview  ***Unique communication styles***  “I mean, I think ... If we don't know them already. You know we're building up a relationship with them and …it's surprised me how well that worked for people that haven't met us in person. And then when we would go out and see them in person [and], they would know who we were because they'd seen us on the screen so, yeah, I mean, I do think it's been a lot better than any of us could have ever predicted and not that's still not ideal, but it's definitely got its place.” E004 HCP interview  ***Trust in what is being seen and heard.***  “There is a gentleman, a wheelchair [user with], quite a profound physical disability. So we went to see him. I crouched down beside him. Said hi. Oh, you're having a cup of tea because I could see he was having a cup of tea. Is that your favourite drink? You couldn't do that the same online as he wouldn't be having his cup of tea. And you take him from the environment and things he's familiar with. And he's not going to be seen familiar stuff. I'm just a head in a box.” E011 HCP interview  ***Rapport between professional groups***  “It's definitely more difficult to build a rapport, and also you don't know the carers” E006 HCP interview  ***Building Trust***  “I mean, rapport is so much easier in the room. You know, I and that and that can be as simple as spotting that they've got a really nice pair of socks on and asking where did you get those socks? Those are amazing. I need some socks like that.”  This participant goes on to say, “I was pleased that it worked better than I thought it would, and especially how it improved over time.” E008 HCP interview  ***HCPs relationships with families***  “So I think it's a very good tool even to talk with the carers or partners, family members. You'll get the whole family in the whole setup and all the information [about] the person and environment in one go with the video consultation. So I think it's a very good tool.” D002 HCP interview  “…there are definite advantages if you want family members to be involved who maybe couldn't come, you know, often we've got family members that live far away. And so it does. It enables them to join in with appointments.” E004 HCP interview |
| **People with Intellectual Disabilities** | **Family Members & Support Workers** | **General Practice & Community Healthcare professionals** |
| **Theme 4. Prepare and personalise** | | |
| ***Being prepared (before)***  “…. And trying to find a bit of knowledge, a bit of trust in them, and know all about their needs and dislikes and do like.” D026 Patient interview  They flag, flag it up on their screen. And some people don't understand. But what doctors and nurses don't understand. About other people's needs and capacity. They do sometimes do know and they don’t know. For they’ve got to flag it up on their screens to know a lot about them.” D026 Patient interview  ***Being prepared (during)***  “Just explain what you're going to be talking about. And make sure that we, they can easily explained everything. D024 Patient interview  **Recurring advice to HCPs**  *“Get to know who they are.”* D023 Patient interview  ***Awareness of Reasonable Adjustments***  “I mean, I go into some of my appointments and they know I never go in by myself, because of my issues and they look at me when I walk into the room and I say “[name of brother]” [to call him along] and [they are] like, hang on. “What's he doing here?” And I go, “well, he's my carer of course he has to be here.” D024 Patient interview  “These unclear few sentences may have impacted the patient as she wipes her eyes in what looks like an annoyance. Her smile goes away” Obervation 3 Integrated Fieldnotes | ***Being prepared (before)***  ***“***So if I was ringing somebody up, I'd be looking at the individual's notes beforehand on the computer and trying to guesstimate or estimate. You know what that problem might be before trying to and it should really be on it. It should be on eyes. That's notes that I'm his advocate or (father’s name) is his advocate, and that they never come to the surgery unaccompanied, they can't express their needs, they’re non-verbal, I mean, hello.” A003 FM PwPID interview  ***Family Frustration with lack of preparedness***  “And when (PwPID name) had a really bad… a really bad seizure. We were rung up by an doctor's … and she was insisting that she talk to (PwPID name). I kept saying to her, please look at his notes. He's non-verbal. He can't express his needs. I need to speak on his behalf and after about 3 attempts she then backed down the allowed me to talk to her. And it was frustrating.” A003 FM PwPID interview  ***Awareness of Reasonable Adjustments***  But what I what I would probably say is , do a thorough, so go through the case notes really thoroughly to see if there's any sort of mitigating circumstances as to why either situation will be better, whether it's face to face or, or virtual… cause 'cause if it will vary that person to person, it won't be the same for everybody. D002 FM interview | ***Being prepared (before Observation)***  “Multiple calls were needed to assist the nurse in these activities.” Observation 2 Fieldnotes  “The nurse had not met the patient before and did not know much about them” Obervation 2 Fieldnote  ***Contingency planning***  “So what we tend to do is kind of have a bit of an agreement. ...[Have] ..an agreement with somebody beforehand, like if I lose you, it's just the connection goes, I need to be able to have a phone number of where you are so that I can ring [you]”. (E007 HCP) AND “…. So we just had the thing that I would basically ring the client's mobile phone and we would carry on as if we hadn't left off. Do you know what I mean? We would just continue with the session as we were. Appreciating that again, a phone call wasn't as good, but it was better to continue with our session and complete the session rather than to keep stopping. And it it appeared it was a kind of fairly frequent issue for them, so it was worth us having that as a contingency.” E007 HCP interview |
| **People with Intellectual Disabilities** | **Family Members & Support Workers** | **General Practice & Community Healthcare professionals** |
| **5. Continue connection** | | |
| ***Feeling connected***  “I mean the thing more that helped because I'd already met her face to face. … So I knew who I was talking to, so it might have been more nervous if I hadn't known who it was that I was going to have on the screen….I knew it was happening. I knew who it was and we just had a normal chit chat and she's got my sense of humour.” D002 Patient interview  **Loss of connection and quality**  “I think things get skipped over this way and especially on the phone they're not as thorough.” B016 Home Manager interview  ***Avoid interruptions***  “Probably the doctor not getting interrupted by the receptionists and other doctors” D023 Patient interview.  “It also didn’t help being on hold quite a few times to get, to let them get information about my meds. D023 Patient interview  ***Post-VC information in accessible formats***  “Yes, I was kind of like jotting notes down as we were going through the review.” D023 Patient interview  “And it makes you have better memory of it, doesn't it? I think that's worth having in writing as well. Yeah, to go back on.” B013 Patient interview | ***Ensuring continuity***  “I think a lot of them like continuity of care. So they like to see the same person and they know it like going to the surgery to see say, I know that some of them have a particular doctor that they would prefer to see and that they enjoy going to say. But I think it's done on a patient need basis I'd imagine.” D028 CP interview  ***Connecting long-distance families/Support Workers***  “It [VC] enables us to maintain contact with long-distance families. You're able to still have quite a person-centred meeting with someone and discuss things and it's in a safe environment. But it's quicker, it's easier. Actions can be followed quicker. Yeah, I do. I do think it's beneficial.” B010 HM interview  ***Mixed experiences of connection and community***  “I think maybe sometimes it [VC] can be a bit more impersonal. It's, you know, if you can suppose if you're with someone, you can gauge their whole surroundings rather than just, you know, sort of seeing the top of the head and that's it.” A004 FM PwPID interview  “I think you know we lost so much of that in COVID we lost that ability to be able to have a community and talk and have a cuppa and just sort of you know.” A002 FM PwPID interview  ***Minimising interruptions***  “Well, it can be noisy and you do get interrupted” B013 CP interview  “…staff members [in assisted living space] occasionally joined in the conversation to quickly help answer queries they knew the answers to.”  Obervation 1 Fieldnote interview  ***Helpfulness of post-VC information***  “They'll [HCP] then send up a follow-up e-mail with the information in that I would normally then print and then be able to relay to the staff team” B010 Home Manager interview | ***Ensuring continuity***  “... and once we get to liking it and do even offer the same [person] every year because they like consistency if they're used to something and we like it, it's better if we keep doing the same one. So, I think that would be my suggestions. Make sure you choose the right person”. D027 HCP interview  ***Continuity service ethos***  “As a service, try to kind of have a bit of a rule of thumb that we'll see everybody during the course of a piece of work. We see everybody in-person at least one time, and that once you've kind of gathered some of that information that you might get from having them into you [clinic] or you going out to them, then you might continue it online.” E007 HCP interview  “…use of ending statements about what will happen next, what they need to do (if anything at all) and the timeframe for the next appointments…”  Observations 1&3 Integrated Fieldnotes  ***Importance of family members/Support Workers*** ***in maintaining connection***  “It [VC] can also mean that somebody can join for part of the consultation and then sort of more easily choose to do their own thing. Whilst either a family carer or paid worker and perhaps working in a residential services, stuff continues. I can make a couple of people who have worked where it's felt really important and they've wanted to be part of the consultation but not necessarily wanted to be present for all of it.” E010 HCP interview  ***Potential for fragmented care, the unintentional consequence of VCs***  “I have seen some adults where I've just given them A-frame and another colleague has then gone out and set them up with the frame and I've never met them. So I. And and you lose that connection. And so they weren't involved, really at all in deciding what frame what they do, because it's all done through carers.” E006 HCP interview  “I think that's really important and my worry is that we are, as a society we the necessity that grew from COVID is being seen as routine and the potential for us to forget what it's like to engage, face to face as human beings. One to one, I think we we have the potential to lose that. You know people live in social media worlds quite often and at the at, you know potentially at the risk of neglecting their face to face relationships. You know the importance of of having people that you can hold and you can hug and you can really sit and have a cry with. We're losing that, I think.” E008 HCP interview  ***Consulting space and interruptions***  “…[it would] be really good to have it somewhere that is kind of nice and quiet so that you're not having all those other interruptions going on in the background.” B010 HCP interview  “I usually conduct this private consultation in a private area… So you try to make it clear that in advance, alright, we need to do it in your private room. Make sure you lock the door. Make sure you are not interrupted or disturbed and try to make sure no noise around so this is part of the preparation…” D001 HCP interview  “I think it's harder to keep people in the room as long on a on a virtual consult…. whenever I see anybody for a follow up appointment, I put aside an hour and you know I do that because it means that we've got flexible time.” E008 HCP interview  “Well, it can be noisy and you do get interrupted” B013 CP interview  ***Importance of post-VC information***  “I think it is important … regardless of whether it's a face to face or a virtual consultation to provide the person with a summary of what's happened that's successful to them as far as is possible.” E010 HCP interview |
| **Abbreviations**  **PwID- People with Intellectual Disabilities**  **PwPID- People with profound intellectual Disabilities**  **HCP- Healthcare Professionals**  **SW- Support Workers**  **FM- Family Members**  **Ob-Observation** | | |

**Weblink to 7 freely available resources to support virtual consultations for people with learning disabilities:** [Virtual consultations for people with learning disabilities, their families and healthcare providers: a co-design study to aid implementation in everyday practice | University of Surrey](https://www.surrey.ac.uk/research-projects/virtual-consultations-people-learning-disabilities-their-families-and-healthcare-providers-co-design)
